# A comparison of manual and automated neural architecture search for white matter tract segmentation

**DOI:** 10.1038/s41598-023-28210-1

**Published:** 2023-01-28

**Authors:** Ari Tchetchenian, Yanming Zhu, Fan Zhang, Lauren J. O’Donnell, Yang Song, Erik Meijering

**Affiliations:** 1 Biomedical Image Computing Group, School of Computer Science and Engineering, University of New South Wales (UNSW), Sydney, NSW Australia; 2 Brigham and Women’s Hospital, Harvard Medical School, Boston, USA

**Keywords:** Computer science, Neuroscience

## Abstract

Segmentation of white matter tracts in diffusion magnetic resonance images is an important first step in many imaging studies of the brain in health and disease. Similar to medical image segmentation in general, a popular approach to white matter tract segmentation is to use U-Net based artificial neural network architectures. Despite many suggested improvements to the U-Net architecture in recent years, there is a lack of systematic comparison of architectural variants for white matter tract segmentation. In this paper, we evaluate multiple U-Net based architectures specifically for this purpose. We compare the results of these networks to those achieved by our own various architecture changes, as well as to new U-Net architectures designed automatically via neural architecture search (NAS). To the best of our knowledge, this is the first study to systematically compare multiple U-Net based architectures for white matter tract segmentation, and the first to use NAS. We find that the recently proposed medical imaging segmentation network UNet3+ slightly outperforms the current state of the art for white matter tract segmentation, and achieves a notably better mean Dice score for segmentation of the fornix (+ 0.01 and + 0.006 mean Dice increase for left and right fornix respectively), a tract that the current state of the art model struggles to segment. UNet3+ also outperforms the current state of the art when little training data is available. Additionally, manual architecture search found that a minor segmentation improvement is observed when an additional, deeper layer is added to the U-shape of UNet3+. However, all networks, including those designed via NAS, achieve similar results, suggesting that there may be benefit in exploring networks that deviate from the general U-Net paradigm.

## Introduction

White matter tract segmentation is the task of delineating the anatomical white matter tracts of a given subject. This information can be useful in a variety of contexts including studies of the brain in health and disease (Parkinson’s:^1^, Alzheimer’s:^2^), and pre-surgical planning^3,4^. It can also be used to help aid tractography, the process of generating 3D models of a brain’s white matter tracts, by creating tract-specific boundaries to guide tractography algorithms ^5^. This paper focuses on direct volumetric tract segmentation, where diffusion-weighted MRI (DWI) voxels are directly labelled according to the anatomical white matter tracts that pass through them.

A variety of approaches for direct white matter tract segmentation exist (see Zhang et al., 2022^6^ for a review). TRACULA^7^ is one popularly used approach, using probabilistic tractography constrained by anatomical priors to generate volumetric probability distributions for white matter tracts. These probabilities are thresholded at 20% of the maximum value of each tract’s distribution to generate voxel-wise white matter tract segmentations. However, TRACULA may result in underestimation of the spatial extent of white matter tracts^8^.

While TRACULA uses T1-weighted MRI scans for deriving anatomical priors, another approach is to directly use anatomical information via tractogram registration. Bundle-Specific Tractography (BST)^8^ is one such method, using a set of template streamlines to define the spatial extent of each white matter tract. The template streamlines are mapped to the subject space, and DWI voxels are labelled according to which white matter tracts have streamlines passing through them. Although BST results in improved spatial coverage compared to prior methods^8^, the quality of the segmentations is entirely dependent on how accurately the template streamlines capture the geometric variability of white matter tracts.

Wasserthal et al. (2018)^9^ instead take a deep learning approach to white matter tract segmentation. Rather than registering a fixed set of template streamlines to the DWI volume of a new subject, a deep network, referred to as TractSeg, is trained to take DWI-derived fibre orientation distribution peaks as input, and directly output segmentations for 72 tracts. TractSeg^5^ is the current state of the art for direct white matter tract segmentation, and is both faster and more accurate than comparable methods, such as TRACULA and a variety of registration-based methods^5,9^. It uses a U-Net architecture incorporating deep supervision^10^, achieving a Dice score of 0.85 on a set of semi-automatically generated reference segmentations^5^.

Little research has been done into improving TractSeg’s segmentation network. In terms of altering the network architecture, Dong et al. (2019)^11^ incorporated a second input branch for T1-weighted MRI data, and demonstrated a statistically significant Dice score increase of 0.005. Rather than altering the network, Lu et al. (2021)^12^ experimented with pre-training the TractSeg network using related pretext tasks. They found that a fine-tuned network trained on pretext tasks achieved a statistically significant 0.189 higher Dice score compared to a baseline TractSeg model when the training set consisted of only 5 labelled tract segmentations. More recently, Lu et al. (2022)^13^ proposed a transfer learning method that reduced the number of annotated examples needed to fine-tune a pre-trained TractSeg model to segment a new white matter tract. Finally, a method proposed by Liu et al. (2022)^14^ achieved state of the art segmentation accuracy, especially in scenarios of lower data quality, by projecting voxel labels to a lower-dimensional space. The TractSeg network was modified to predict 36 labels per voxel, and an additional network component was introduced to map this 36-element vector back to the original 72-element label space.

However, rather than manually altering the TractSeg network to achieve better segmentation performance, another potential approach is to use neural architecture search (NAS). NAS is a relatively new research domain focusing on the task of automatically searching for an optimal deep network architecture, with a seminal paper from Zoph and Le in 2016^15^ showing state of the art results in image classification and language modelling. Although methods to perform NAS drastically vary, in general, networks are designed by defining a search space, search strategy, and performance estimation strategy^16^. Various NAS approaches have been shown to outperform manually designed networks across a variety of medical imaging modalities, including MRI and CT segmentation^17–19^. In this paper, we use the NAS approach proposed by Zhu and Meijering (2021)^20^, which achieved high segmentation performance across a variety of cell segmentation datasets. We chose this method as it uses a fixed U-Net macro-structure for the architectures of all searched models, while using NAS to optimise the internal composition of each layer of the network’s U-shape. This ensures that searched models do not deviate from the well-established U-Net architecture paradigm, hence allowing for a direct comparison of U-Net models searched by NAS to various existing U-Net based models.

In this paper, we compare the performance of deep networks designed via our own manual experimentation, and automated changes to a base U-Net model via NAS, for the purpose of white matter tract segmentation. For a baseline comparison, we train multiple, previously published, state of the art U-Net based medical image segmentation networks. We then manually adjust the best performing network for peak segmentation performance by experimenting with network depth, skip connections, loss functions and convolutional operations. We finally compare the segmentation performance of the base and adjusted state of the art networks to a NAS approach to architecture design. To the best of our knowledge, this is the first study to systematically compare multiple U-Net based architectures for white matter tract segmentation, and the first to use NAS for this segmentation task.

## Material and methods

### Dataset

We use the dataset published in Wasserthal et al. (2018)^9^, referred to as the TractSeg dataset, for white matter tract segmentation (available at https://doi.org/10.5281/zenodo.1285152). This dataset provides tractograms for 72 white matter tracts for 105 subjects of the WU-Minn Human Connectome Project (HCP)^21^. These tractograms are semi-automatically generated by combining existing tractography methods and related algorithms with manual error correction^9^. It is the largest publicly available dataset of tract-specific tractograms generated from human subjects. The creation of the WU-Minn HCP dataset from which these tractograms were generated was approved by the institutional review board of Washington University in St. Louis (IRB #201204036). Informed consent was given by all subjects to the Human Connectome Project consortium^21^. We use tractograms from the TractSeg dataset, alongside DWI and T1-weighted MRI scans from HCP dataset. Our use of all HCP data conformed to the Open Access Data Use Terms (available at https://www.humanconnectome.org/study/hcp-young-adult/document/wu-minn-hcp-consortium-open-access-data-use-terms).

Our task is to train models that take fibre orientation distribution peaks as input, and output white matter tract segmentations. We will now briefly describe the data generation process and details of the input and output data. A more detailed description can be found in Wasserthal et al. (2019)^5^.

#### Fibre orientation distribution peaks

The input to all models is a 2D image with 144 × 144 voxels and 9 channels. These 9 channels are the concatenation of three 3D vectors corresponding to the three most common orientations of fibres passing through each voxel. These three vectors are called fibre orientation distribution (FOD) peaks, and are computed by applying multi-shell multi-tissue constrained spherical deconvolution (CSD)^22^ to DWI scans, using T1-weighted MRI scans as an additional source of information. In this study, these DWI and T1-weighted scans are from the WU-Minn HCP dataset (see “Data availability” section for access details).

The HCP DWI scans are 3D volumes of size 145 × 174 × 145 voxels, and hence, CSD generates 3D peaks volumes rather than 2D peaks images. As in Wasserthal et al. (2019)^5^, we obtain 2D peaks images by cropping the 3D peaks volume to 144 × 144 × 144 voxels, without loss of any non-background data, and stepping through the volume in coronal, sagittal, and axial orientations to get 2D peaks images of shape 144 × 144 × 9 voxels. We specifically chose to work with 2D data to allow for direct comparisons of our work to previous work published on TractSeg, as well as due to the benefit of reduced GPU memory footprint that enables deeper architectures to be experimented with.

#### White matter tract segmentations

As in Wasserthal et al. (2019)^5^, all models generate tract segmentations for 72 white matter tracts, concatenated channel-wise into a segmentation mask of size 144 × 144 × 72 voxels. For a full list of anatomical tracts, see Wasserthal et al. (2018) ^9^. Using code from the TractSeg GitHub repository (https://github.com/MIC-DKFZ/TractSeg), the ground-truth segmentation volumes used for model supervision are generated from the TractSeg dataset’s tractograms. This is achieved by setting voxels to 1 if at least one tractogram streamline runs through them, and 0 otherwise. The same cropping and slicing approach applied to the 3D peaks volume is used to generate these 2D segmentation masks from the 3D segmentation volumes.

### Deep network architectures

We train and compare the segmentation performance of five existing state of the art U-Net based architectures. These architectures capture a variety of approaches to improving segmentation performance, including the use of deep supervision, dense connections, attention, and a large number of skip connections. All networks take a 144 × 144 × 9 peaks image as input, and generate a 144 × 144 × 72 segmentation mask.

#### U-Net

U-Net (Supplementary Fig. S1) is a fully-convolutional encoder-decoder deep network with skip connections between corresponding encoding and decoding nodes^23^. The encoder nodes generate high-resolution features that are combined with the upsampled features of the decoder via skip connection concatenation^23^. U-Nets have been used extensively in medical imaging segmentation across a wide variety of data modalities, including MRI^9,24^, microscopy^23^, X-ray^25^ and ultrasound^25,26^.

#### U-Net with deep supervision (DS-U-Net)

Wasserthal et al. (2019)^5^ used a modified version of U-Net that was initially designed for brain tumour segmentation^10^. This network introduces deep supervision to U-Net’s decoding path (Supplementary Fig. S2), which uses an additional 2 convolutions at the second and third decoding node concatenation blocks. The resulting feature maps are summed element-wise with the network’s final output to achieve supervision of these two deep decoding nodes. U-Net with deep supervision (DS-U-Net) is the current state of the art segmentation network for the TractSeg dataset, and is used as a baseline for comparing the performance of other models in this paper.

#### UNet++ 

UNet++ (Supplementary Fig. S3) introduces sequences of interconnected convolutional blocks within the skip connections of U-Net^27^. Zhou et al. (2018)^27^ describe these blocks as nested dense convolutional blocks, and rationalise this design decision as an attempt to increase semantic similarity between the encoding and decoding feature maps. In its introductory paper, UNet++ was shown to outperform U-Net in cell nuclei segmentation, colon polyp segmentation, liver segmentation, and lung nodule segmentation^27^.

#### Attention U-Net

Attention U-Net^28^ adds an attention gate to each of U-Net’s skip connections (Supplementary Fig. S4). The proposed attention gate is a ‘soft-attention’ gate that weights different parts of the feature maps according to learned attention weights. Oktay et al. (2018)^28^ observed that attention gates allow the network to focus on target structures of varying shapes and sizes. They also demonstrated that Attention U-Net outperformed U-Net in multi-class CT abdominal segmentation.

#### UNet3+ 

UNet3+ ^29^ introduces additional skip connections to each decoding node of U-Net (Supplementary Fig. S5). This involves every decoding node receiving skip connections from all lower-level decoding nodes, and all equivalent and higher-level encoding nodes. Huang et al. (2020)^29^ state that this approach allows for the network to leverage more information than UNet++ from the multiple scales of features available throughout the various encoding and decoding nodes of the network. They showed that UNet3+ outperforms U-Net and UNet++ in liver and spleen segmentation tasks ^29^.

### Training details

All models were trained for 250 epochs. The Adamax^30^ optimiser was used with the learning rate decaying by a factor of 0.1 when validation loss did not decrease for 20 epochs. The learning rate was selected for each model independently based on validation performance during a preliminary training run of 10 epochs (see Supplementary Table S1 for the learning rate of each network). A batch size of 47 was used for all models to match results reported in Wasserthal et al. (2019)^5^, as well as due to GPU memory limitations.

As our models work with 2D data, 2D slices are extracted from the 3D dataset items by stepping through each 3D volume in sagittal, coronal and axial orientations. As in Wasserthal et al. (2019)^5^, during training, slices are randomly sampled from these orientations so that a single network can operate with data sliced from any orientation. Each input slice is normalised by subtracting its mean and dividing by its standard deviation. Following normalisation, the data augmentation strategy reported by Wasserthal et al. (2019)^5^ was used, which applies elastic deformation, rotation, zooming, displacement, resampling, and Gaussian noise to each training sample (see Supplementary Table S2 for a list of data augmentation parameters). We altered this approach to assign each augmentation type a 20% independent chance of being applied to a given training sample, as preliminary experimentation found that this improved performance.

The model output is then fed into a sigmoid function to map voxel values to probabilities. During validation and evaluation, a threshold of 0.5 is applied to the sigmoid function’s output, with background pixels being set to 0 and foreground pixels to 1. Finally, binary cross-entropy (BCE) loss is computed after application of the sigmoid function to the network output. The loss is computed between the output of the sigmoid function and the ground truth segmentation mask that corresponds to the peaks image that is input to the network. We experimented with replacing BCE with other loss functions described in the “Further network tuning” section.

Training was performed using PyTorch on a National Computational Infrastructure (NCI Australia) node. Our configured environment contained 16 GB of memory, 12 24-core Intel Xeon Cascade Lake processors, and an Nvidia V100 GPU with 32 GB of memory.

### Further network tuning

A variety of successfully established and recently proposed loss functions, as well as various architecture alterations, were investigated to observe the impact of manual design changes on segmentation performance. These further experiments were only performed on the best performing unaltered architecture due to computational limitations.

The investigated loss functions are described in the following list. Note that for the following equations, *L* is the loss between the ground truth *y* and the model output *x*.

- Equation (1) defines BCE, which is a well-established loss for binary classification tasks.
  1 $$L_{BCE} = y \cdot logx + \left( {1 - y} \right) \cdot log\left( {1 - x} \right)$$
- Eq. (2) defines focal loss^31^, which modifies cross-entropy loss to place a higher loss burden on miss-classified examples. We use an α of 0.25 and γ of 2, where *A* is α for pixels where the target class is 0, and 1 – α for pixels where the target class is 1.
  2 $$L_{FL} = A \cdot (1 - e^{{ - L_{BCE} }} )^{\gamma } \cdot L_{BCE}$$
- Eq. (3) defines Dice loss^32^, which computes the Dice score between the segmentation output and ground truth. We use an $\in$ of 1.
  3 $$L_{DL} = 1 - \frac{2xy + \in }{{x + y + \in }}$$
- Eq. (4) defines perimeter loss^33^, which adds an extra term that measures error at the perimeter of a segmentation mask, to an existing loss function such as BCE or Dice loss. For this existing loss function we use BCE, and a λ of 0.01. *L*_perimeter_ is the mean square error between the output and target segmentation region contours. As described in Jurdi et al. (2021)^33^, the contours are calculated using the difference between min and max pooling.
  4 $$L_{PL} = \left( {1 - \lambda } \right) \cdot L_{BCE} + \lambda \cdot L_{perimeter}$$

We also investigated the following architecture changes, which alter the network architecture without deviating greatly from the general U-Net design paradigm:

- Skip connections: We experimented with removing all skip connections.
- Network depth: We experimented with varying the number of encoding/decoding nodes in the network. Models of depth 2, 3, 4, 5 and 6 were trained (see Fig. S5 for the depth 5 architecture diagram, and Supplementary Figs. S6-9 for depths 2, 3, 4 and 6).
- Convolution operations: We experimented with replacing all convolution operations of the network. Standard convolution, dilated convolution^34^, and depthwise-separable convolution^35^ were attempted.

### Neural architecture search

**Figure 1:**
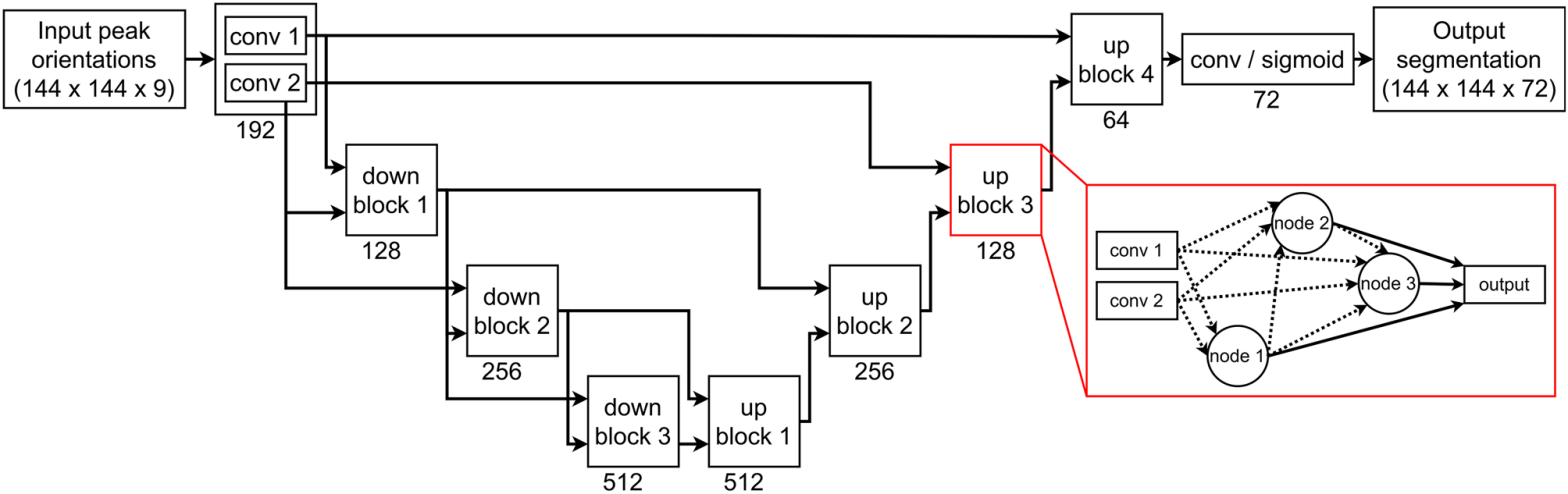
Architecture diagram for the macro-architecture that remains fixed during NAS. The numbers under blocks indicate the number of filters used by each convolution-based operation within the block. The fixed convolution operations in the first block called ’conv 1’ and ’conv 2’ are 1 × 1 stride 1, and 3 × 3 stride 2 convolutions respectively. An example of the inside of a block is highlighted in red. Each block contains two fixed 1 × 1 convolution operations, and 3 nodes each consisting of 2 basic operations. All convolution-based operations within a node will be 3 × 3 with ’same’ padding. The dotted arrows indicate possible connections between nodes, and NAS will select 2 input paths for each node. The output node of an up block concatenates its 3 inputs, while the output node of a down block contains 3 basic operations preceding the concatenation.

We compared the previously described architectures based on U-Net to networks that were automatically designed via NAS. We used the NAS method introduced by Zhu and Meijering (2021)^20^, which demonstrated consistently high performance across a variety of cell segmentation datasets, more so than any other existing method. This NAS method uses a predefined U-like macro-architecture with skip connections (Fig. 1), and applies NAS to select basic operations (BOs) within each encoding and decoding block, as well as the connections between these BOs within a given block. We refer to these BOs and their connections as the micro-architecture of the network. These BOs consist of different types of convolutions, pooling methods, and normalisation operations. The search algorithm involves alternating between fixing the kernel parameters and optimising the architecture parameters, and vice versa, where kernel parameters are optimised using a training set, while architecture parameters are optimised using a separate validation set. See Zhu and Meijering (2021)^20^ for more details concerning the BOs and search algorithm. The size of the training, validation, and test sets are the same for both NAS and non-NAS methods; a detailed description is given in the “Evaluation” section.

To remain consistent with the previously described U-like networks, we used 64 filters for each convolution operation in the first block of the U-like macro-architecture, and doubled the number of filters for each deeper block in the U-like shape. Each block contained 3 nodes, each with a fixed number of BOs during training, which were pruned to the best 2 BOs on final architecture selection. Both the kernel and architecture optimisers used the Adam algorithm^30^, each with a learning rate of 3 × 10^−5^. A batch size of 47 was used. NAS was stopped after 80 epochs due to computation time limitations on the system used for training; however, the validation Dice score curve had essentially plateaued (see Supplementary Fig. S10). Once the architecture search was completed, the architecture with the highest validation set Dice score was re-trained using the methodology described in the “Training details” section.

To observe the benefit of NAS compared to an arbitrary network using the same macro-architecture, we evaluated models using the same macro-architecture but with randomly assigned and connected BOs within the allowable set of BOs and connections.

### Evaluation

#### Methodology

All models were evaluated using fivefold cross validation with a 60/20/20 training/validation/testing split. In the same manner as Wasserthal et al. (2019)^5^, this was achieved by assigning 63 subjects to the training set, 21 to the validation set, and 21 to the test set. In the case of NAS, the architecture search was repeated for each of the 5 folds, but the validation set for each fold contained no overlap with the validation sets of other folds since the validation set is the only data used by the architecture optimiser of our NAS method. The resulting 5 architectures were then re-trained and evaluated.

The key evaluation metric was the Dice similarity coefficient (DSC). For a given subject, each model generated 144 2D segmentation masks. The DSC between the generated volume and the ground truth was computed for each of the 72 tracts individually. The final score for the subject was the mean of these 72 Dice scores. This was repeated for each slicing orientation (sagittal, axial, coronal) and the final score for a subject was the mean of these 3 scores. The final score for a model was then the mean over all 21 test set subjects.

We also computed the relative volume difference (RVD) using the same averaging approach. RVD reports the absolute difference in volume between the generated and ground truth volumes, as a fraction of the ground truth volume. Hence, it is a size-based segmentation metric, which we used to supplement the DSC, an overlap-based segmentation metric^36^.

The epoch with the highest Dice score on the validation set was selected for the final evaluation. During validation, this score was computed as the mean DSC of all 2D slices, while during evaluation on the test set the slices for a subject were first concatenated into a 3D volume before DSC calculation. This difference was due to the extra memory and time required for the concatenation step, hence excluding it during validation made training much more efficient.

#### Statistical analysis

The statistical significance of model differences was evaluated using the two-tailed Wilcoxon signed-rank test^37^ with Bonferroni correction to correct for multiple comparisons (α = 0.05/*n*, *n* = number of model comparisons). Differences between models were computed at the tract level; hence across all folds of cross-validation, the Dice scores (and RVD values) for each of the 72 tracts of all 105 subjects were compared, resulting in 7,560 Dice score differences (and RVD value differences) for each model comparison.

## Results and discussion

We experimented with a large variety of state of the art network architectures, and various manual adjustments to UNet3+ . For a summary of the architecture and training parameter variations we experimented with, along with the associated mean Dice score and mean RVD values, see Supplementary Table S3.

### Performance of state of the art networks

**Figure 2:**
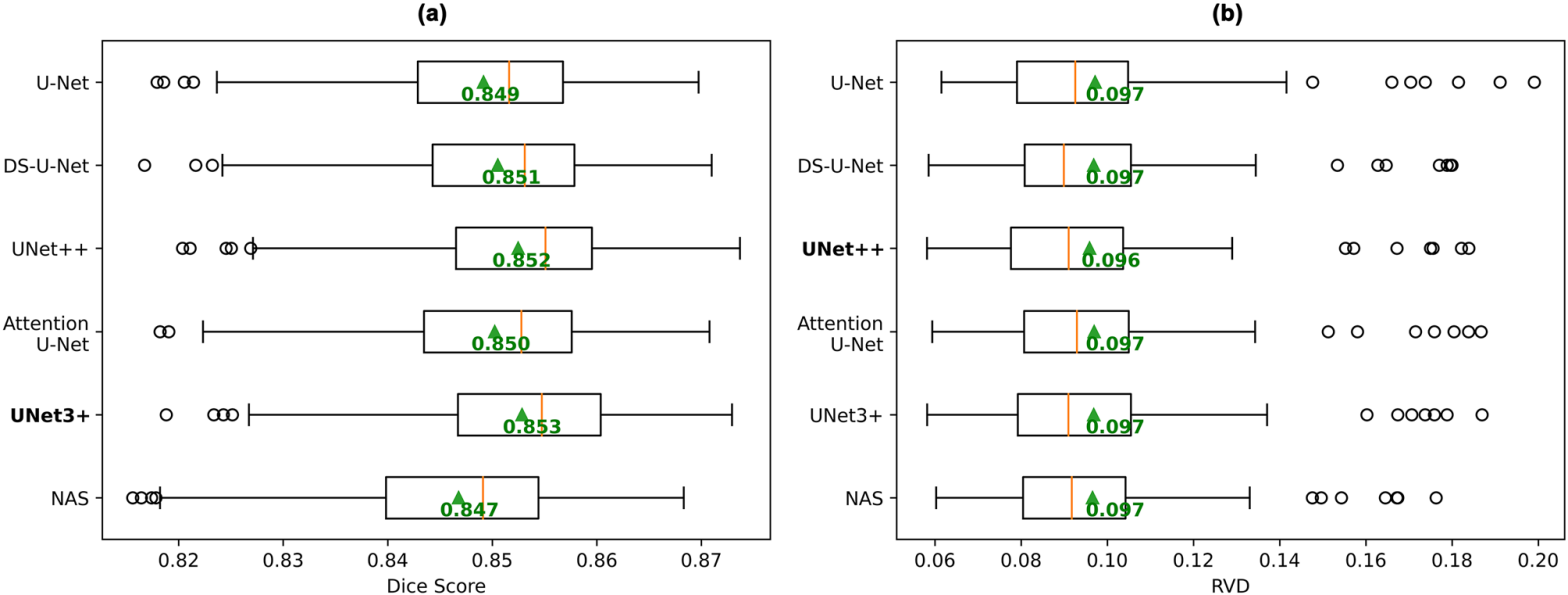
Comparison of performance of six different models. Box plots of (**a**) the mean Dice scores for all subjects (higher is better), and (**b**) the mean RVD values for all subjects (lower is better). Mean across all subject scores is indicated by green triangle and text. Orange bar indicates median score. Models with the highest mean Dice score and lowest RVD value are bolded.

All existing state of the art networks (U-Net, DS-U-Net, UNet++ , Attention U-Net, UNet3+) achieved similar mean Dice scores (Fig. 2a). However, the mean Dice score difference between any pair of models was statistically significant (*p* < 0.05/15) except for the difference between DS-U-Net and Attention U-Net (*p* = 0.008).

UNet++ and UNet3+ both outperformed the current state of the art model DS-U-Net, with UNet3+ performing the best with a 0.002 mean Dice score improvement over DS-U-Net, while also only requiring approximately 74% (27.2 M) of the parameters of DS-U-Net (37.1 M) and UNet++ (36.7 M).

All models achieved mean RVD values of 0.097 (Fig. 2b), except for UNet++ which achieved 0.096. Although this improvement in RVD was statistically significant (*p* < 0.05/15 for all model comparisons with UNet++), the effect size of 0.1% absolute improvement was very small.

### Manual network tuning

**Figure 3:**
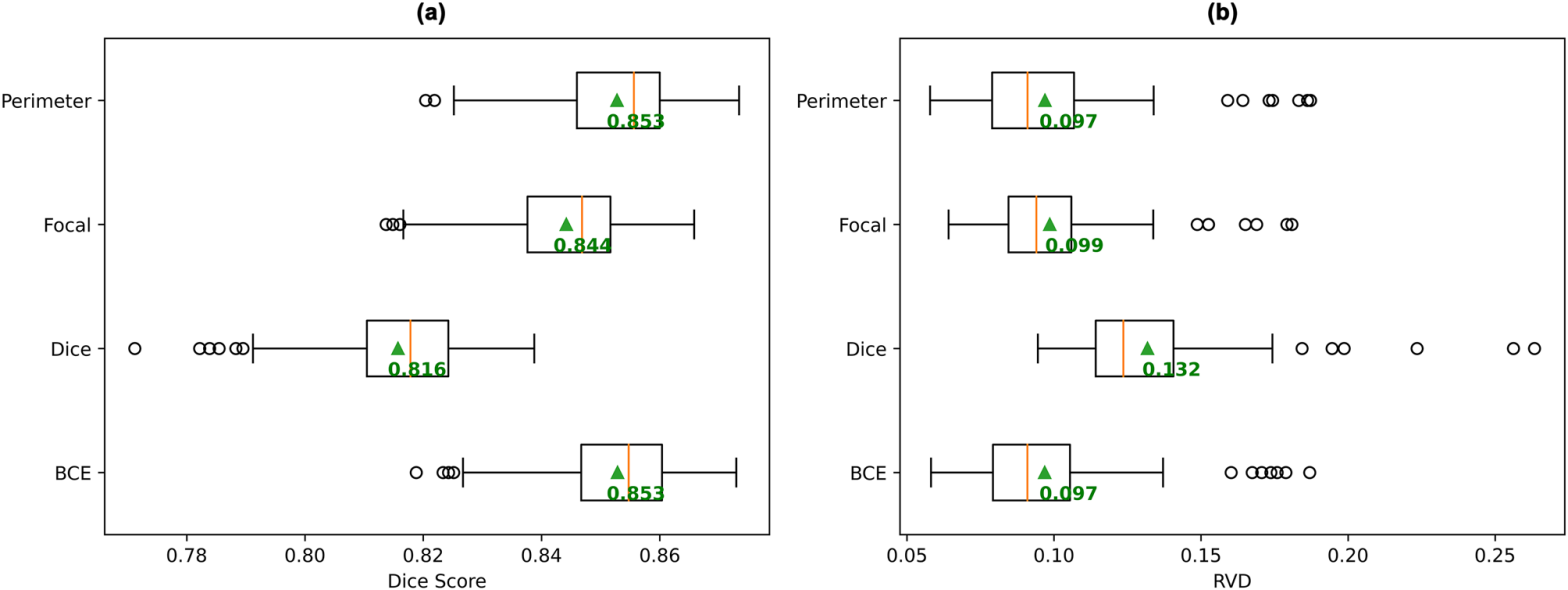
Comparison of performance of UNet3+ trained with different loss functions. Box plots of (**a**) the mean Dice scores for all subjects (higher is better), and (**b**) the mean RVD values for all subjects (lower is better). Mean across all subject scores is indicated by green triangle and text. Orange bar indicates median score.

Manual tuning of UNet3+ produced minimal improvement over the standard UNet3+ model. However, the impact of these changes on UNet3+ performance highlighted the relative importance of these model and training parameters for the use of UNet3+ for the task of white matter tract segmentation. One such finding was that changing the loss function had a relatively large impact on UNet3+ performance (Fig. 3). Perimeter loss was found to perform just as well as the standard BCE loss, with no statistically significant difference in their mean Dice scores (*p* = 0.218) or mean RVD values (*p* = 0.590), while both focal loss and Dice loss performed notably worse than BCE and perimeter loss.

**Figure 4:**
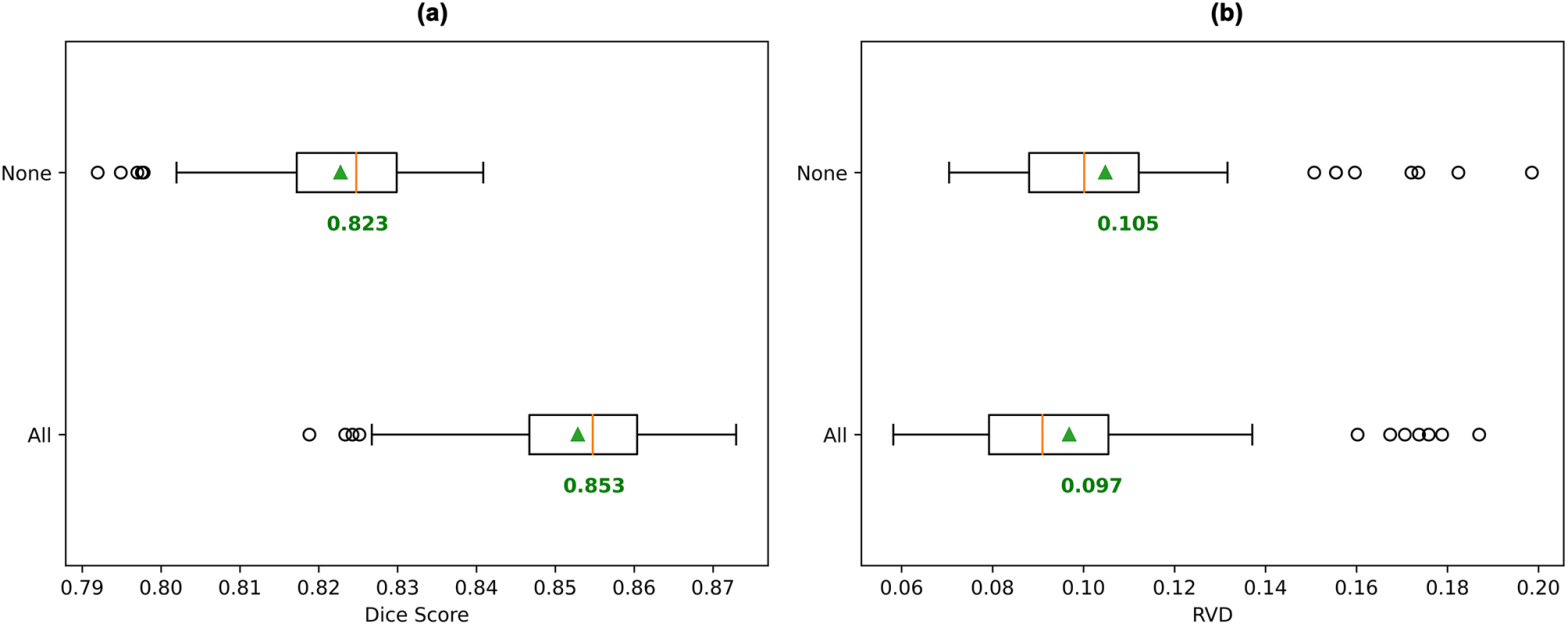
Comparison of UNet3+ trained with and without skip connections. Box plots of (**a**) the mean Dice scores for all subjects (higher is better), and (**b**) the mean RVD values for all subjects (lower is better). Mean across all subject scores is indicated by green triangle and text. Orange bar indicates median score.

As expected, skip connections were found to be important for UNet3+ in the task of white matter tract segmentation, as removing skip connections resulted in a statistically significant drop in mean Dice score of 0.03 (*p* = 0) and increase in mean RVD of 0.008 (*p* = 0) (Fig. 4) with the model using skip connections achieving a higher mean Dice score and lower mean RVD for every tested subject. However, it is surprising that a U-Net framework with no skip connections was able to achieve approximately 96% of the mean Dice score and an only 8% higher mean RVD value of an unaltered UNet3+ with all skip connections. The purpose of skip connections, as explained in the original U-Net paper^23^, is to assist high-resolution segmentation by combining high-resolution features computed during the encoding stage of the network with the features computed during the decoding stage. Hence, the unexpectedly small segmentation quality drop when removing skip connections may indicate that the input data can be encoded into compact features, which can be decoded without information from additional high-resolution features via skip connections. More broadly, the relatively low importance of these high-resolution features from the encoder, suggests that high resolution image features of DWI scans have a relatively low impact on white matter tract structure and shape.

**Figure 5:**
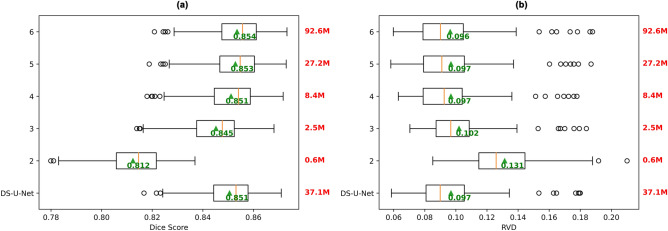
Box plots of performance of UNet3+ models with different depths (2–6), with DS-U-Net as a baseline for comparison. The depth of the network corresponds to the number of encoding and decoding nodes in the U-shape of the network. Number of parameters (in millions) for each model is depicted in red text. Box plots of (**a**) the mean Dice scores for all subjects (higher is better), and (**b**) the mean RVD values for all subjects (lower is better). Mean across all subject scores is indicated by green arrow and text. Orange bar indicates median score. See Fig. S5 for the depth 5 architecture diagram, and Supplementary Figs. S6-9 for depths 2, 3, 4 and 6.

We also found that UNet3+ performance increased as the depth of the network increased (Fig. 5). UNet3+ with depth 6 achieved the highest mean Dice score of 0.854, a 0.001 improvement over the unaltered UNet3+ architecture, and a 0.003 improvement over the state of the art DS-U-Net. However, there was no statistically significant difference between the corresponding mean RVD values (*p* = 0.513, *p* = 0.241). Additionally, increasing the depth of the network resulted in drastically larger model size, with the depth 6 model having 92.6 million trainable parameters compared to the 27.2 million trainable parameters of the UNet3+ network of depth 5. Hence, further increasing the network depth beyond a depth of 6 would not be feasible. Diminishing returns were also observed, with minute differences in performance between models of depth 4, 5 and 6. Notably, there is a relatively large drop in mean Dice score and increase in mean RVD value when going from depth 3 (2.5 million trainable parameters) to 2 (0.6 million trainable parameters). However, even with a depth of 2 the model achieves a mean Dice score of 0.812 and mean RVD value of 0.131, suggesting that the present white matter tract segmentation task may not require a complex model to achieve decent performance.

**Figure 6:**
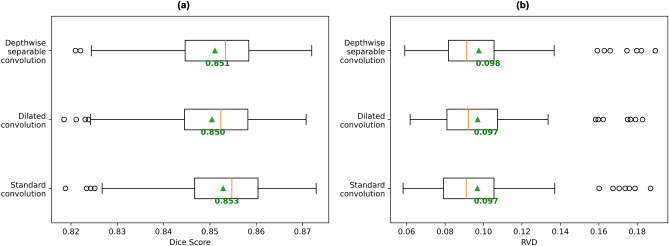
Box plots of performance of UNet3+ models with different convolution operations. All convolution operations in the network were replaced by the specified convolution operations. Box plots of (**a**) the mean Dice scores for all subjects (higher is better), and (b) the mean RVD values for all subjects (lower is better). Mean across all subject scores is indicated by green arrow and text. Orange bar indicates median score.

Regarding convolution operations, standard convolution outperformed depthwise separable convolutions and dilated convolutions (Fig. 6a) in mean Dice score. Dilated convolutions have a larger receptive field over standard convolution operations ^34^. Since they did not show an increase in performance over standard convolution for the present task, it implies that the receptive field of the standard convolutions are sufficient when using the UNet3+ architecture. On the other hand, depthwise separable convolutions reduced the number of parameters from 27.2 M (for standard convolution) to 3.2 M, but also suffered a minimal mean Dice drop similar to dilated convolutions, and achieved a 0.001 higher mean RVD value (*p* = 0.0006) (Fig. 6b).

### Comparing DS-U-Net and Unet3+ 

**Figure 7:**
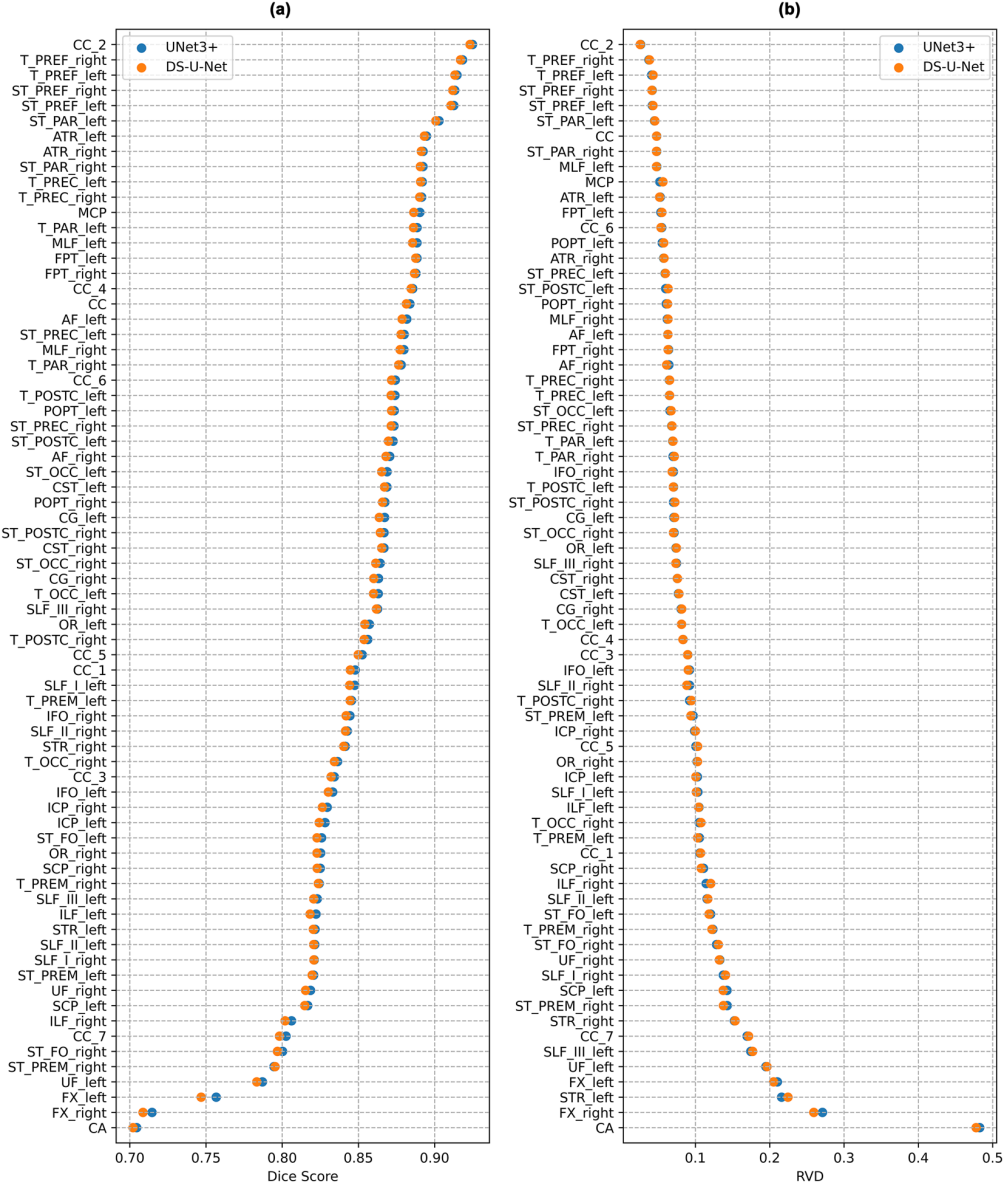
Segmentation performance across all subjects for each of the 72 white matter tracts. (**a**) Mean Dice scores (higher is better), and (**b**) mean RVD values (lower is better). See Supplementary Table S4 for a list of full tract names.

**Figure 8:**
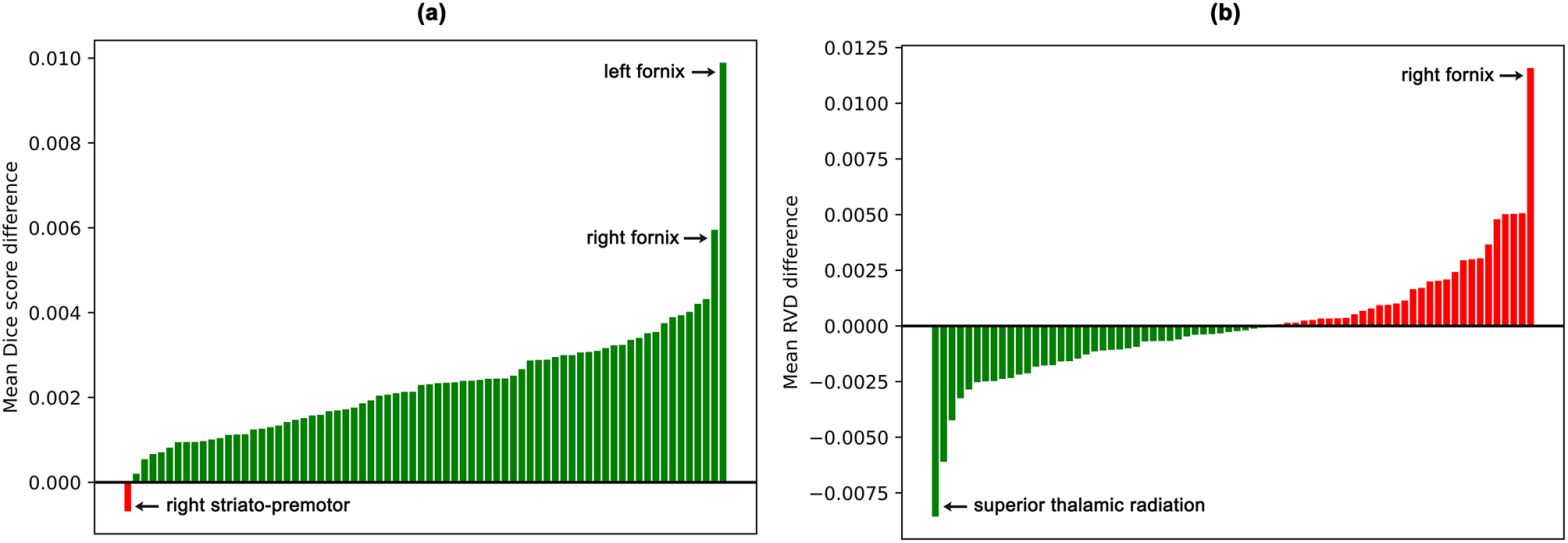
Segmentation performance differences between UNet3+ and DS-U-Net plotted for each of the 72 tracts. (**a**) Mean Dice score differences, where positive scores (displayed in green) indicate superior UNet3+ performance and negative scores (displayed in red) indicate superior DS-U-Net performance. (**b**) Mean RVD value differences, where negative scores (displayed in green) indicate superior UNet3+ performance and positive scores (displayed in red) indicate superior DS-U-Net performance.

DS-U-Net and Unet3+ perform very similarly for the majority of tracts. However, it is clear from our results (Fig. 7) that the majority of the difference in performance is for the worst performing tracts. We found that Unet3+ achieved superior mean Dice scores for these more difficult tracts (Fig. 7a). One such tract was the fornix, which had a notably higher mean Dice score (+ 0.01 and + 0.006 for left and right fornix respectively) when segmented via Unet3+ compared to the state of the art DS-U-Net architecture (Fig. 8a). However, there was no clearly superior model in terms of mean RVD values for the worst performing tracts (Fig. 7b). For example, DS-U-Net achieved a notably better mean RVD value for the second worst performing tract (right fornix), while Unet3+ achieved a notably better mean RVD value for the third worst performing tract (left superior thalamic radiation) (Fig. 8b).

**Figure 9:**
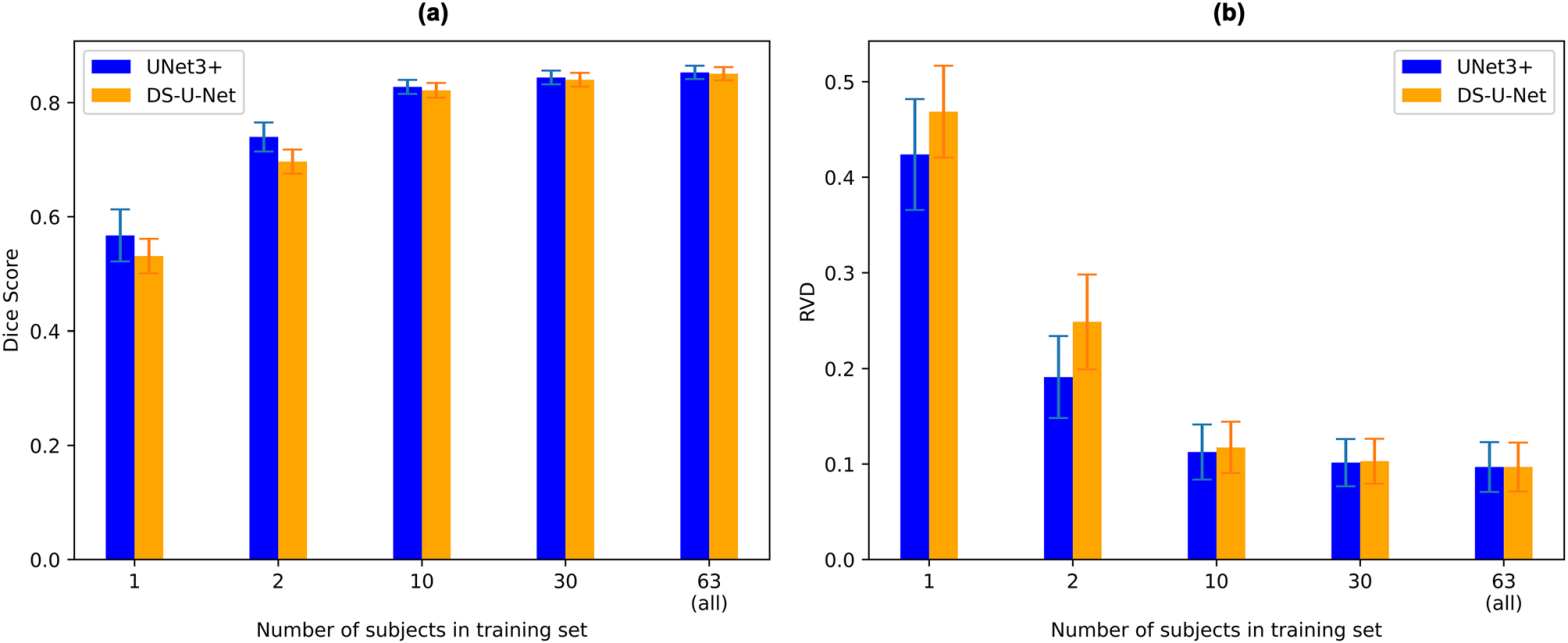
Segmentation performance of UNet3+ and DS-U-Net with different numbers of training subjects. (**a**) Mean Dice score across all subjects and all tracts (higher is better), and (**b**) mean RVD value across all subjects and all tracts (lower is better). Error bars indicate standard deviation for the 105 subject scores.

Another notable difference between DS-U-Net and Unet3+ is that Unet3+ performs considerably better when less training data is available. Directly comparing the performance of the two architectures when they are trained with 1, 2, 10, 30, and all 63 subjects (Fig. 9), it is clear that Unet3+ performs notably better than DS-U-Net when the training set consists of 1 or 2 subjects, with the difference in performance diminishing as more subjects are added to the training set. Additionally, it is surprising that mean Dice scores over 0.7 and mean RVD value of below 0.2 can be achieved when only two reference subjects are available at training time. This implies that either inter-subject variability is small, or that the deep models can generalise quite easily.

**Figure 10:**
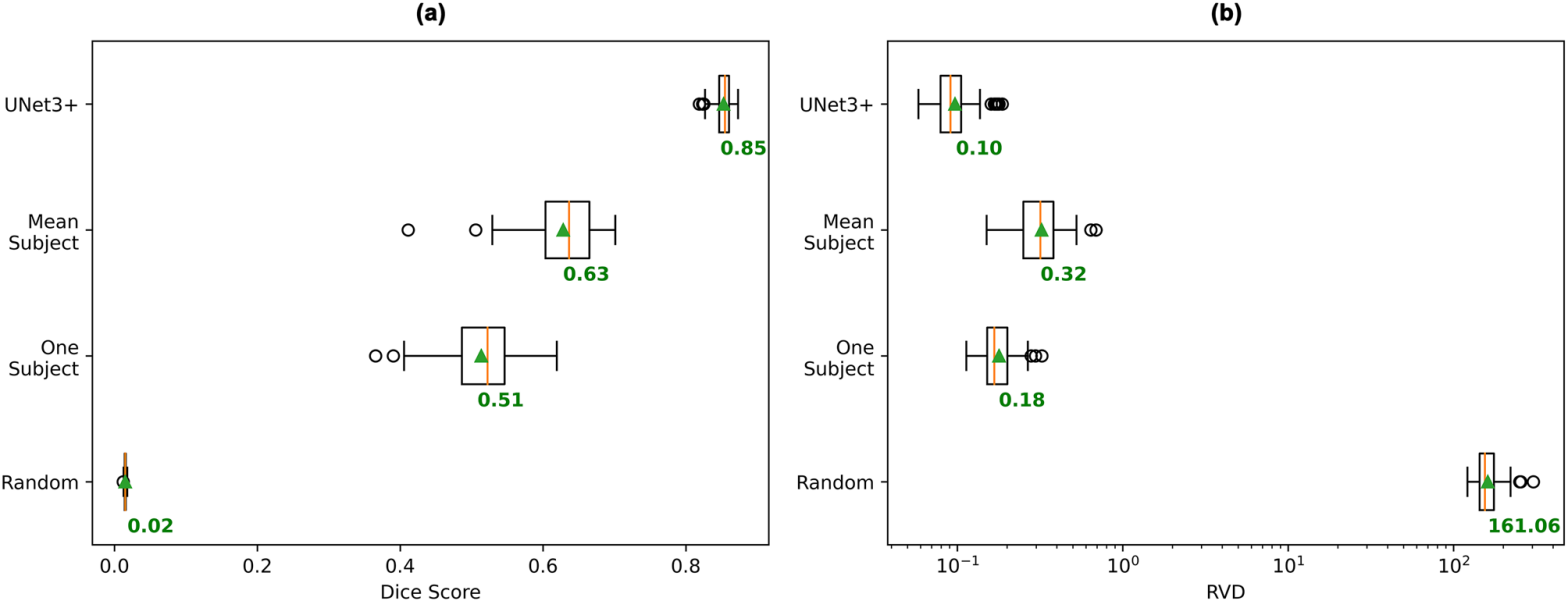
Performance comparison of various segmentation approaches. Box plots are computed on the (**a**) Dice scores (higher is better), and (**b**) RVD values (lower is better), across all 105 subjects, where the score for a subject is the mean across its 72 tract scores. Mean across all subject scores is indicated by green triangle and text. Orange bar indicates median score. ‘Random’ indicates a model that always generates a segmentation volume of uniform random data in the range [0,1] that is thresholded at 0.5. The ‘One Subject’ model takes the ground truth of a single subject and will always output that subject’s segmentation volume as the model output (voxels thresholded at 0.5). ’Mean Subject’ model will take the mean across all segmentation volumes in the training set and output it as the model output (voxels thresholded at 0.3).

To test whether a deep model is needed at all, we evaluated a few, much simpler, segmentation methods (Fig. 10) using the same cross-validation test sets described in the “Evaluation” section. For a baseline, we calculated the Dice scores when our model always outputs a segmentation mask consisting of uniform random data (range = [0,1]) thresholded at 0.5. This model performed barely above a mean Dice score of 0, with a mean RVD value over 160. The next model ‘One Subject’ took the segmentation volume from a 1 subject ‘training set’ and always output the ground truth segmentation volume for this subject. This achieved a considerably better mean Dice score of 0.51, and mean RVD value of 0.18. This result can be directly compared to the previously described performance of UNet3+ that was trained on a single subject, which achieved a mean Dice score of 0.57, and mean RVD value of 0.42. Hence, by introducing a deep model, the mean Dice score improved by around 0.06 points, while the mean RVD value worsened by around 0.24 points.

To measure the impact of inter-subject variability on segmentation performance, our final experiment was to take the mean of the segmentation volumes of all items in the training set, and always output the resulting volume. If inter-subject variability was sufficiently small, then we would expect the ‘Mean Subject’ model to perform comparably to a fully trained UNet3+ . However, the best performance we were able to achieve via this ‘Mean Subject’ model was a mean Dice score of 0.63 and mean RVD value of 0.32, when the mean segmentation volume was thresholded at 0.3 during evaluation. In comparison, UNet3+ trained on a complete 63 subject training set achieved a mean Dice score of 0.85 (35% higher than ‘Mean Subject’) and mean RVD value of 0.1 (30% of the RVD of the ‘Mean Subject’) This is evidence in support of inter-subject variability being high, and evidence against the hypothesis that UNet3+ trained on two subjects performs so well due to low inter-subject variability.

### Qualitative evaluation of UNet3+ 

**Figure 11:**
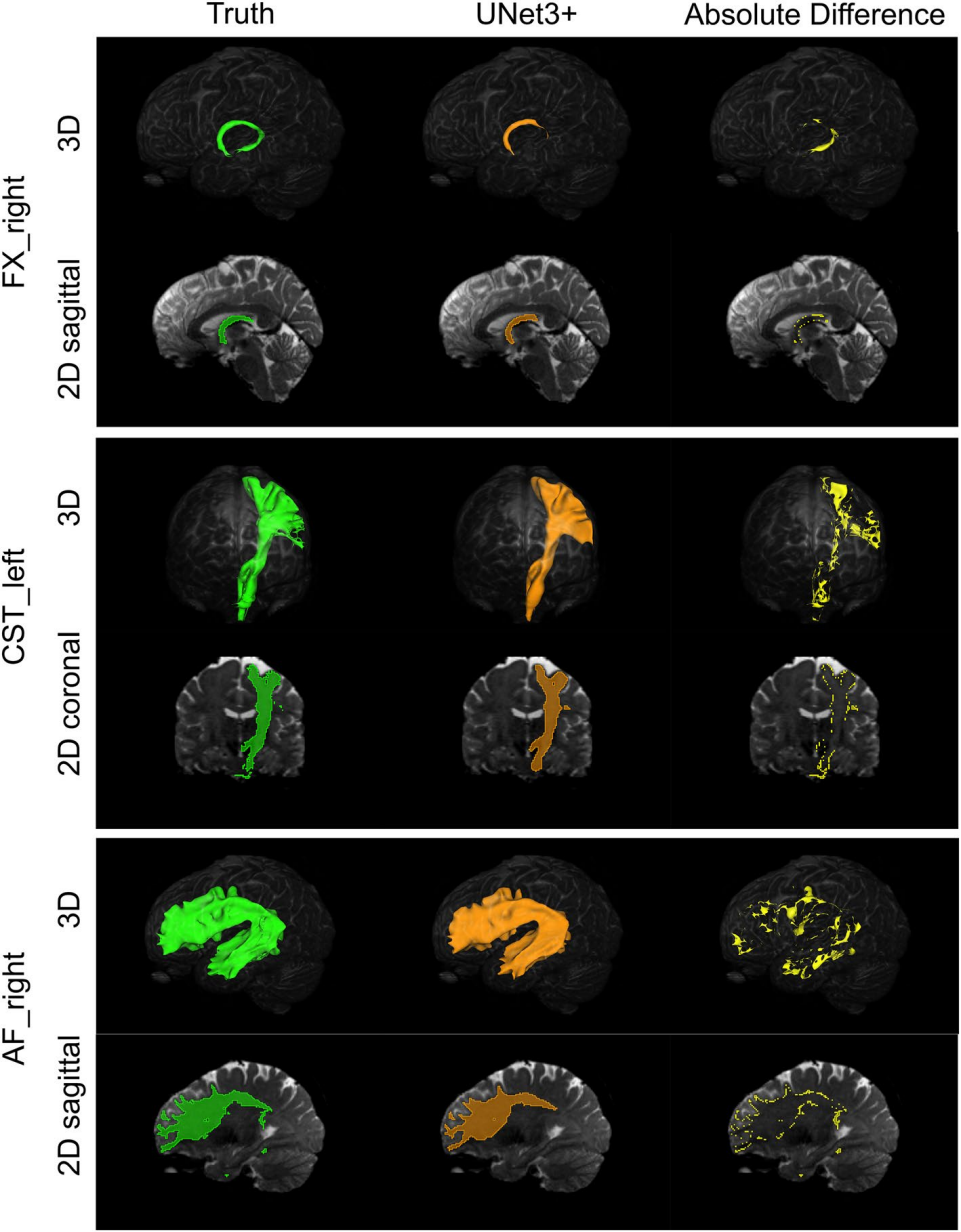
Segmentation output for a random slice of a random test set subject for large (Arcuate Fascicle, AF_right), medium (Corticospinal tract, CST_left), and small (Fornix, FX_right) white matter tracts. ’Truth’ is the ground truth segmentation mask, ‘UNet3+ ’ is the output from a fully-trained UNet3+ model, and ’Absolute Difference’ is the absolute difference between ’Truth’ and ’UNet3+ ’. Segmentations are displayed as both 3D volumes and 2D slices.

We observed (Fig. 11) that essentially all segmentation errors occur at the segmentation region’s perimeter. Although some of this error appears speckled and noise-like, there are also larger regions of error that are unlikely to stem from noisy ground truth data. Given this observation, we would expect that introducing a perimeter loss factor to the loss function would improve these results. However, as we reported in the “Manual network tuning” section, our experimentation with perimeter loss yielded no statistically significant difference compared to standard BCE loss. However, our experimentation was quite simple, using a fixed λ of 0.01 for the perimeter factor in the loss equation. We also experimented with increasing λ over time; however, preliminary results were poor, so this experimentation avenue was stopped. We believe there is promise in future work that explores error reduction at the segmentation perimeter. This may involve more experimentation with the perimeter loss function described in Jurdi et al. (2021)^33^, or perhaps a more complex deep learning approach that adds a second post-processing network that is specifically designed to minimise perimeter errors.

**Figure 12:**
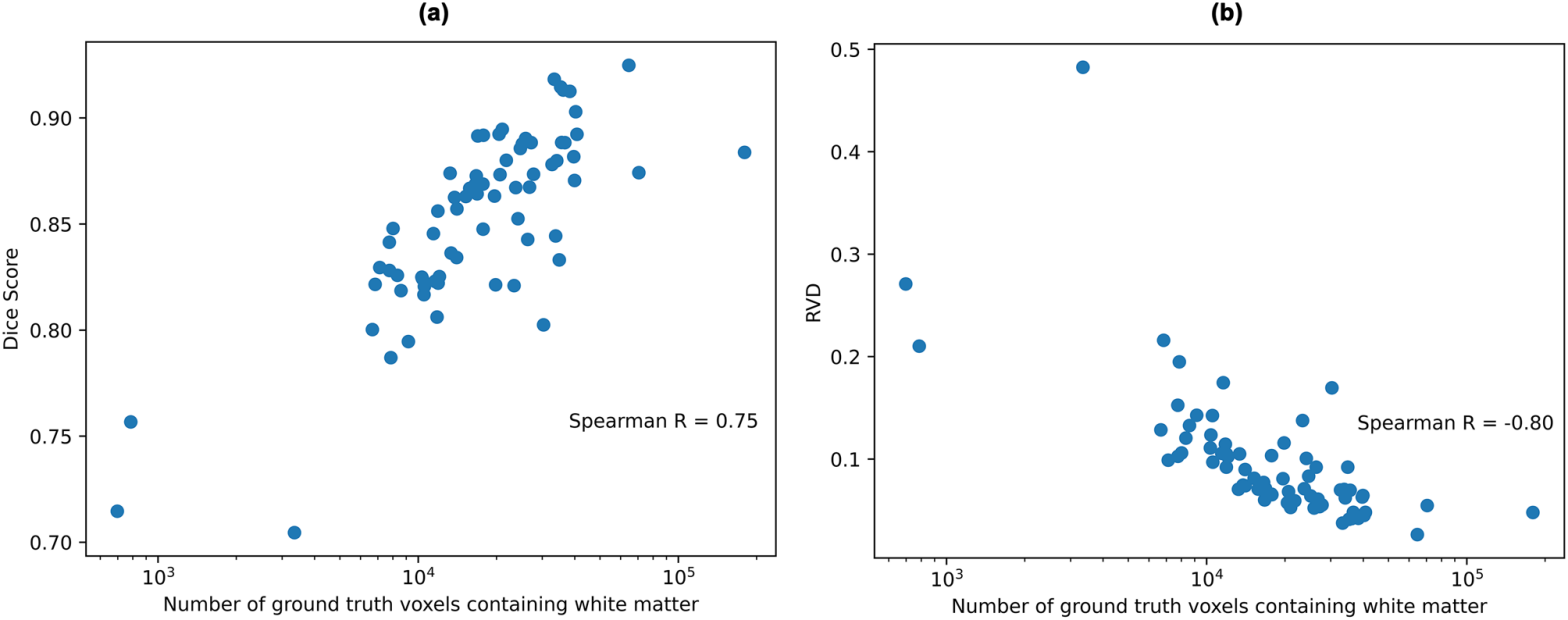
Plot of mean tract size vs. segmentation performance for all 72 white matter tracts. (**a**) Mean Dice score for each tract (higher is better), and (**b**) mean RVD value for each tract (lower is better). Tract size is plotted on a logarithmic axis, and is measured as the number of voxels labelled as containing white matter in the ground truth segmentation mask for a given tract, averaged over all 105 subjects. Each circular marker indicates a white matter tract.

We also observed an expected downward trend in UNet3+ Dice score, and upward trend in RVD as the size of the tract decreases. Plotting the size of each tract’s ground truth volume against the UNet3+ Dice score and RVD value (each averaged across 105 subjects) results in a very clear correlation (Fig. 12). The corresponding Spearman’s rank correlation coefficient is 0.75 for Dice score and -0.80 for RVD. These correlations between tract size and metric scores were expected, as smaller tracts are inherently more difficult to learn segmentations for, due to minor changes to the segmentation volume having relatively larger impacts on loss, as well as both Dice score and RVD, compared to larger tracts. Further work could explore this multi-class segmentation imbalance issue through the use of appropriate loss functions such as those outlined in Sugino et al. (2021)^38^.

**Figure 13:**
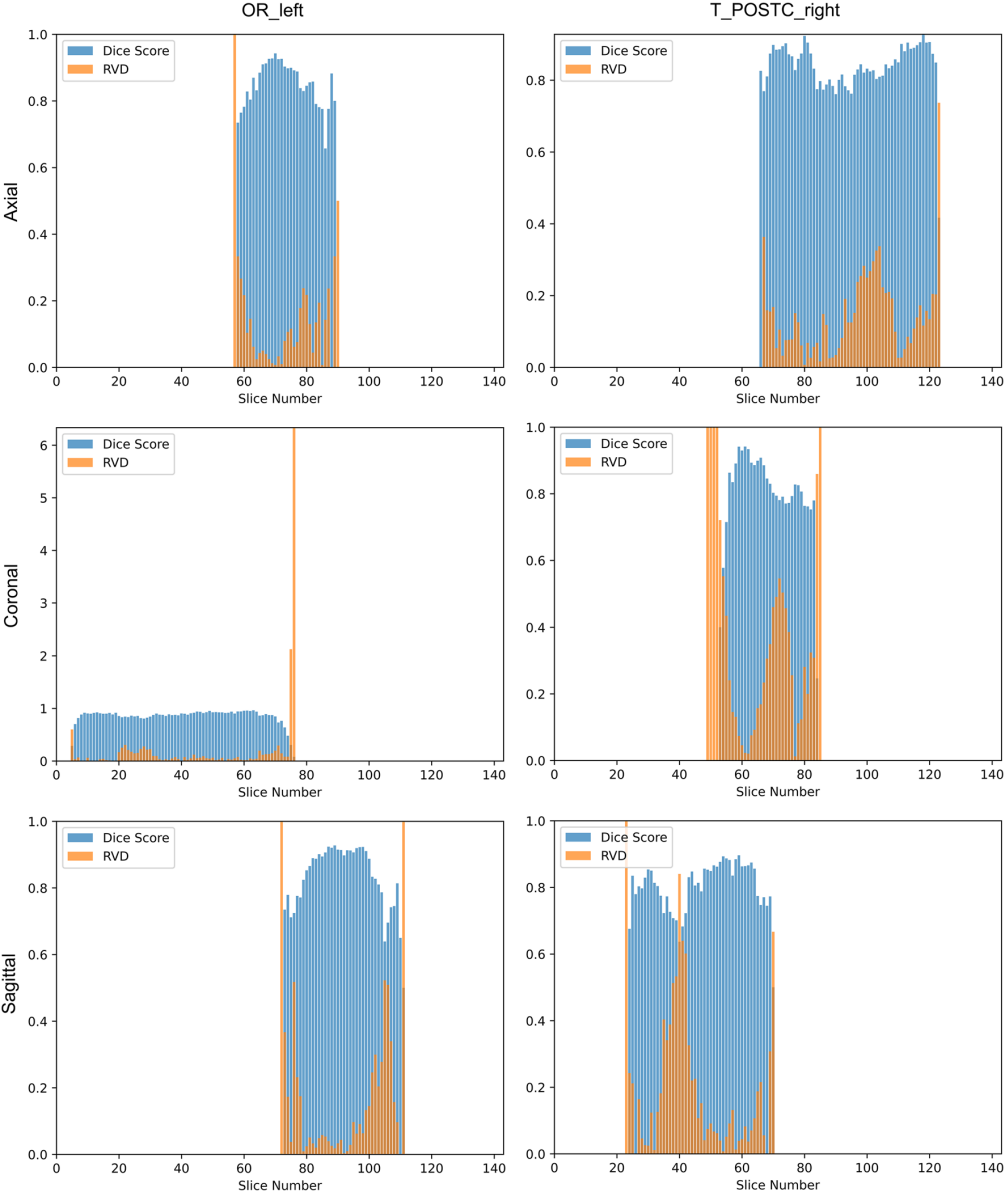
Segmentation performance as a function of slice number. We plot the mean (of all subjects) Dice score (higher is better) and RVD value (lower is better) for each slice number for the 144 × 144 × 144 voxels. This is done for two tracts with close to average performance (approximately 0.85 Dice score): Left Optic Radiation (OR_left) and Right Thalamo-Postcentral (T_POSTC_right), with 3D volumes being sliced in axial, coronal, and sagittal orientations.

We also investigated segmentation performance as a function of slice number by plotting the Dice score and RVD value as we step through the segmentation volume (Fig. 13). Regardless of slicing orientation, performance tends to drop off for the first and last few slices. This is likely due to the first and last few slices having small segmentation masks, resulting in the poor small tract performance issue discussed previously. Alongside the proposed solutions for segmenting small tracts more precisely, future work may address the slice drop-off issue by utilising neighbouring slice data when making predictions for a given slice. A potentially promising network is ConvLSTM^39^, which could integrate past and future slices by introducing recurrent connections to the network.

### NAS

**Figure 14:**
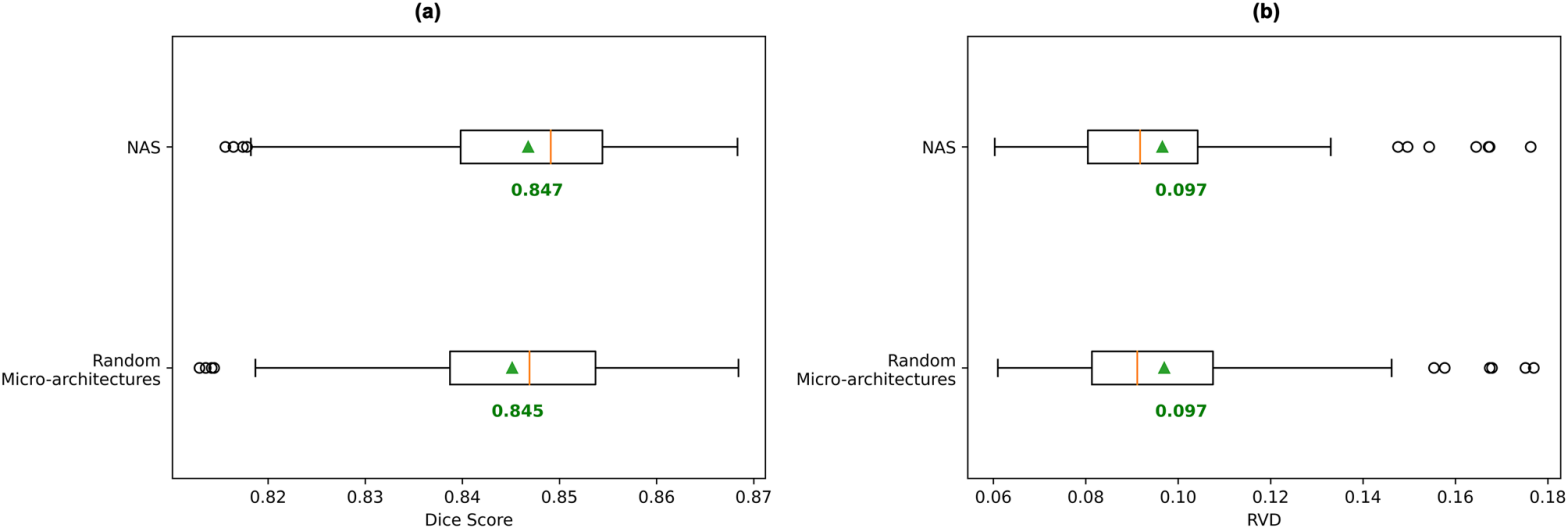
Comparison of networks with micro-architectures designed via NAS and models with randomised micro-architectures. Box plots are computed on the (**a**) Dice scores (higher is better), and (**b**) RVD values (lower is better), across all 105 subjects, where the score for a subject is the mean across its 72 tract scores. Mean across all subject scores is indicated by green triangle and text. Orange bar indicates median score.

NAS performed worse than the other 5 existing network architectures (Fig. 2). One potential explanation for this is the micro-architecture of the network having minimal impact on overall performance. Supporting this explanation, we observed minimal difference in test set mean Dice score, and no difference in test set mean RVD between networks designed via 80 epochs of NAS training and networks with the same macro-architecture but using randomly designed micro-architectures (Fig. 14). From this, we infer that the performance of NAS in our segmentation task is determined largely by network macro-architecture. The NAS approach used in our experimentation uses manually defined macro-architecture parameters, hence we propose that future experimentation concerning NAS for white matter tract segmentation focus on NAS methods incorporating macro-architecture parameters into the search space itself.

Comparing the micro-architectures of the networks designed for each of the 5 folds of cross-validation, we found that the mean difference in basic operations across all pairs of networks was 50%. This difference value is the proportion of basic operations that are different between two architectures, where basic operations are compared based on their location in the network. This difference value is below the 77% mean difference between the 5 random micro-architecture networks, so there is some similarity in the micro-architectures that are being learned, though this similarity is not immediately clear. However, one notable area of similarity was the final down block, which exclusively used identity operations in 4 of the 5 NAS architectures followed by exclusive use of average pooling operations for the down sampling portion of the block. This domination of identity operations suggests that the final down block might not be necessary for the network, further exemplifying the need for future research to explore manipulation of network macro-architecture in more detail.

### The similar performance problem

We have explored a variety of methods to improve segmentation performance, including a variety of existing network architectures, manual adjustments to UNet3+ , and NAS. Although we found that UNet3+ outperformed the state of the art DS-U-Net in a variety of contexts, all network architectures (U-Net, DS-U-Net, UNet++, Attention U-Net, UNet3+ , and NAS) achieved very similar mean Dice scores of approximately 0.85, and mean RVD values of 0.1 (Fig. 2, Fig. 14). One potential explanation may be that the 144 × 144 peaks images that are input to the network do not contain enough information and are therefore limiting the segmentation performance. To verify whether this is the case without needing to produce a higher resolution dataset, future experimentation could consider down-sampling the input data to various degrees, and observing the trend in segmentation performance as the resolution increases to judge whether further increase in input data resolution may improve performance. This was not attempted in this paper due to computational limitations. Super-resolution algorithms applied to the input data, or alternative input formats could also be explored.

Another potential explanation is that all our experimentation has focused on relatively similar macro-architectures. Hence, there may be great value in exploring architectures that drastically differ from the U-Net paradigm. Once such approach are vision transformers, which supplant convolution operations by using patch-based self-attention^40^. Karimi et al. (2022)^41^ found that a vision transformer approach outperformed UNet++ for segmentation of the brain cortical plate, pancreas, and hippocampus.

## Conclusions

Through a thorough exploration of a variety of U-Net architectures, we found that UNet3+ slightly outperformed the current state of the art of DS-U-Net (Wasserthal et al., 2019)^5^ for the task of white matter tract segmentation.

The mean Dice score increase of UNet3+ was more notable in tracts where both DS-U-Net and UNet3+ struggled, in particular the fornix where the mean Dice scores increased by 0.01 and 0.006 for the left and right fornix respectively. UNet3+ also performed considerably better when less training data was available. We also found that UNet3+ performed slightly better when an extra, deeper layer was added. However, UNet3+ still performed decently well when the architecture was modified to reduce complexity, including reduced network depth and removal of skip connections.

Analysing segmentation results in more detail, we found that errors tend to occur at the perimeter of the segmentation regions, as well as the first and last few slices of the segmentation volume. Smaller tracts were also found to be more difficult to segment than larger tracts. We believe that future work will improve overall segmentation performance by experimenting more thoroughly with loss at the perimeter^33^, multi-class segmentation imbalance loss functions^38^, and incorporating past and future slice data to current slice prediction via ConvLSTM^39^ or related networks.

We also found that our NAS method, which automatically designs the micro-architecture of the network, performed relatively poorly, while also achieving minimal improvement over networks with randomly designed micro-architectures. Combining this result with the overall very similar performance across all networks experimented with in this paper, we suggest that performance may be limited by the resolution of the input data, or by the macro-architecture of the network. We propose that future experimentation focus on both of these aspects, perhaps deviating more drastically from the U-Net paradigm.

Finally, our input data was derived from high-quality scans from the HCP dataset, and our segmentation dataset was limited to deep white matter tracts. There would be great value in future work that evaluates the discussed architectures on independent, clinical-quality datasets, or trains and evaluates these architectures to segment superficial white matter tracts.

## Supplementary Information

Supplementary Information.

## Data Availability

DWI and T1-weighted MRI scans used in this study for generating peaks images are from the HCP Open Access portion of the WU-Minn Human Connectome Project 1200 Subjects dataset, publicly available at: https://db.humanconnectome.org/. The data can be accessed after account registration and acceptance of the HCP Open Access Data Use Terms available at https://www.humanconnectome.org/study/hcp-young-adult/document/wu-minn-hcp-consortium-open-access-data-use-terms . We also use tractograms from the TractSeg dataset, publicly available at: https://doi.org/10.5281/zenodo.1285152. All code developed for our experiments is publicly available via GitHub at https://github.com/aritche/white-matter-segmentation.
