# Supplementary material for: A comparison of manual and automated neural architecture search for white matter tract segmentation
Source: Sci Rep. 2023 Jan 28;13:1617. doi: 10.1038/s41598-023-28210-1 (PMC9884270; doi:10.1038/s41598-023-28210-1)
Supplement: Supplementary file 1 — Supplementary Information. [file 41598_2023_28210_MOESM1_ESM.pdf]

A comparison of manual and automated neural architecture search for white matter tract segmentation

Ari Tchetchenian[a,*], Yanming Zhu[a], Fan Zhang[b], Lauren J. O'Donnell[b], Yang Song[a], Erik Meijering[a]

[a]*Biomedical Image Computing Group, School of Computer Science and Engineering, University of New South Wales (UNSW), NSW, Australia*

[b]*Brigham and Women's Hospital, Harvard Medical School, Boston, USA*

* *Corresponding author email: a.tchetchenian@unsw.edu.au*

SUPPLEMENTARY INFORMATION

Model	Learning Rate
U-Net	0.001
DS-U-Net	0.001
UNet++	0.001
Attention U-Net	0.001
UNet3+	0.001
NAS (re-training a fixed architecture)	0.001
UNet3+ (perimeter loss)	0.001
UNet3+ (dice loss)	0.0001
UNet3+ (focal loss)	0.001
UNet3+ (no skip connections)	0.001
UNet3+ (depth 2)	0.001
UNet3+ (depth 3)	0.001
UNet3+ (depth 4)	0.001
UNet3+ (depth 6)	0.001
UNet3+ (dilated convolutions)	0.001
UNet3+ (depthwise-separable convolutions)	0.001

Supplementary Table S1: Summary of learning rates used when training each network.

Augmentation Types	Value Range	Probability of Use
Rotation	$U[-45,45]$	0.2
Elastic Deformation	$\alpha = U[90,120]$, $\sigma = U[9,11]$	0.2
Displacement	$D_x = U[-10,10]$, $D_y = U[-10,10]$	0.2
Zooming	$U[0.9,1.5]$	0.2
Resampling	$U[0.5,1]$	0.2
Gaussian Noise	mean=0, variance=$U[0,0.05]$	0.2

Supplementary Table S2: Summary of data augmentation parameters, where $U[a,b]$ is the random uniform distribution inclusive of a and b.

Model	Parameter Altered	Variant	Mean Dice score	Mean RVD value
U-Net	-	-	0.849	0.097
DS-U-Net	-	-	0.851	0.097
UNet++	-	-	0.852	0.096
Attention U-Net	-	-	0.850	0.097
UNet3+	-	-	0.853	0.097
NAS	-	-	0.847	0.097
	-	Random micro-architectures	0.845	0.097
UNet3+	Loss function	Perimeter loss	0.853	0.097
		Focal loss	0.844	0.099
		Dice loss	0.816	0.132
		BCE loss	0.853	0.097
	Skip connections	None	0.823	0.105
		All	0.853	0.097
	Network depth	2	0.812	0.131
		3	0.845	0.102
		4	0.851	0.097
		5 (default UNet3+)	0.853	0.097
		6	0.854	0.096
	Convolution type	Depthwise separable	0.851	0.098
		Dilated	0.850	0.097
		Standard	0.853	0.097
	Training set size	1 subject	0.567	0.424
		2 subjects	0.740	0.191
		10 subjects	0.827	0.113
		30 subjects	0.844	0.101
		63 subjects (entire training set)	0.853	0.097
DS-U-Net	Training set size	1 subject	0.531	0.469
		2 subjects	0.697	0.249
		10 subjects	0.821	0.117
		30 subjects	0.840	0.103
		63 subjects (entire training set)	0.851	0.097
Always output random uniform data (thresholded at 0.5)	-	-	0.020	161.06
Always output one exemplar subject	-	-	0.510	0.180
Always output the training set mean (thresholded at 0.3)	-	-	0.630	0.320

Supplementary Table S3: Summary of all model architectures and variants, and the associated mean Dice score and mean RVD values.

Tract Name	Abbreviation
Arcuate Fascicle	AF
Anterior Thalamic Radiation	ATR
Anterior Commissure	CA
Corpus Callosum - Rostrum	CC_1
Corpus Callosum - Genu	CC_2
Corpus Callosum - Rostral body	CC_3
Corpus Callosum - Anterior midbody	CC_4
Corpus Callosum - Posterior midbody	CC_5
Corpus Callosum - Isthmus	CC_6
Corpus Callosum - Splenium	CC_7
Corpus Callosum - All	CC
Cingulum	CG
Corticospinal Tract	CST
Fronto-Pontine Tract	FPT
Fornix	FX
Inferior Cerebellar Peduncle	ICP
Inferior Occipito-Frontal Fascicle	IFO
Inferior Longitudinal Fascicle	ILF
Middle Cerebellar Peduncle	MCP
Middle Longitudinal Fascicle	MLF
Optic Radiation	OR
Parieto-Occipital Pontine	POPT
Superior Cerebellar Peduncle	SCP
Superior Longitudinal Fascicle I	SLF_I
Superior Longitudinal Fascicle II	SLF_II
Superior Longitudinal Fascicle III	SLF_III
Striato-Fronto-Orbital	ST_FO
Striato-Occipital	ST_OCC
Striato-Parietal	ST_PAR
Striato-Postcentral	ST_POSTC
Striato-Precentral	ST_PREC
Striato-Prefrontal	ST_PREF
Striato-Premotor	ST_PREM
Superior Thalamic Radiation	STR
Thalamo-Occipital	T_OCC
Thalamo-Parietal	T_PAR
Thalamo-Postcentral	T_POSTC
Thalamo-Precentral	T_PREC
Thalamo-Prefrontal	T_PREF
Thalamo-Premotor	T_PREM
Uncinate Fascicle	UF

Supplementary Table S4: Full names of white matter tracts, and the corresponding abbreviation used in this paper.

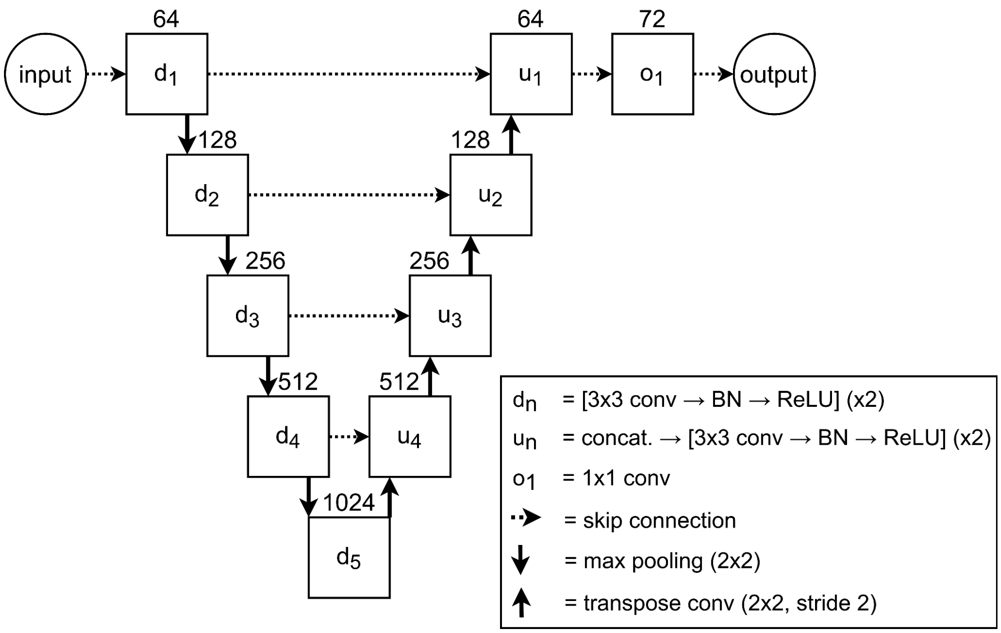

Supplementary Figure S1: U-Net model architecture. Each u_n node concatenates its inputs channel-wise before applying convolution operations. Number above each node indicates the number of filters for all convolution operations within that node, and (if applicable) the number of filters used by the transpose convolution that is feeding into that node. All convolutions use 'same' padding. BN indicates batch normalisation, and ReLU indicates rectified linear unit activation.

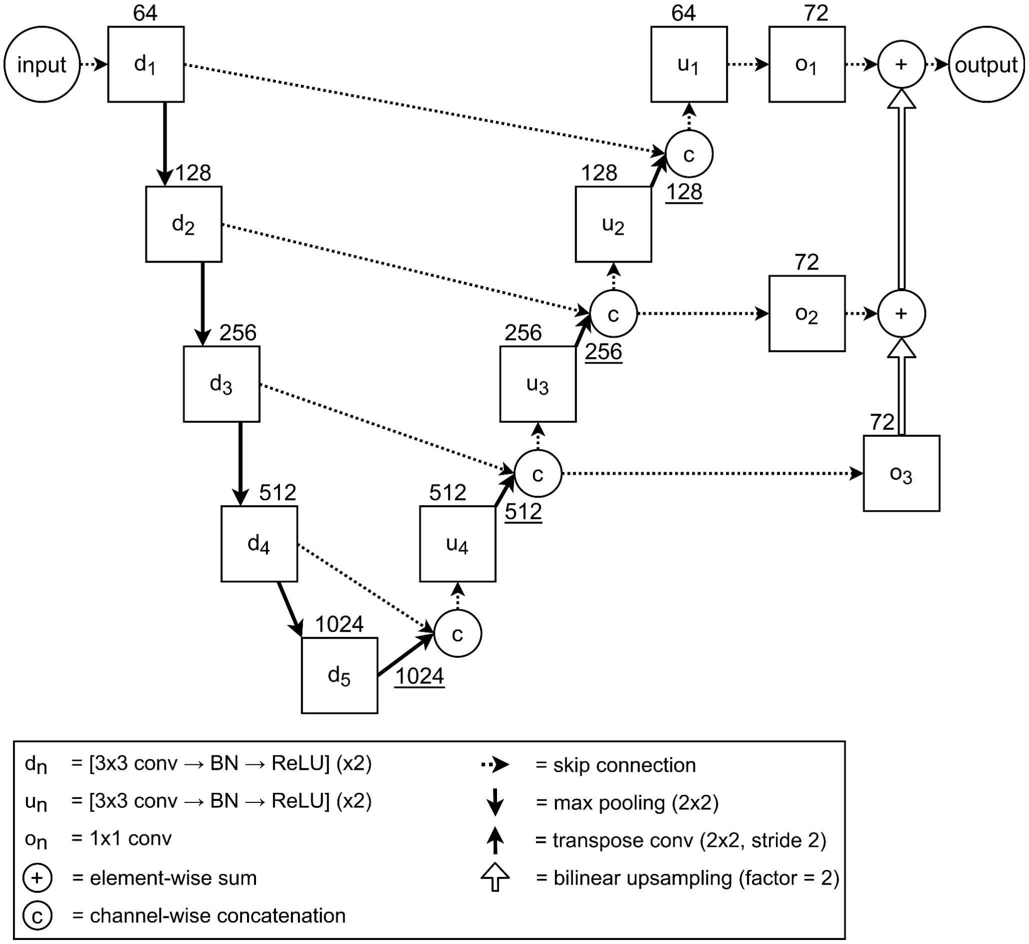

Supplementary Figure S2: DS-U-Net model architecture. Number above each node indicates the number of filters for all convolution operations within that node. Number of filters used by transpose convolutions are indicated in underlined text. All convolutions use ’same’ padding. BN indicates batch normalisation, and ReLU indicates rectified linear unit activation.

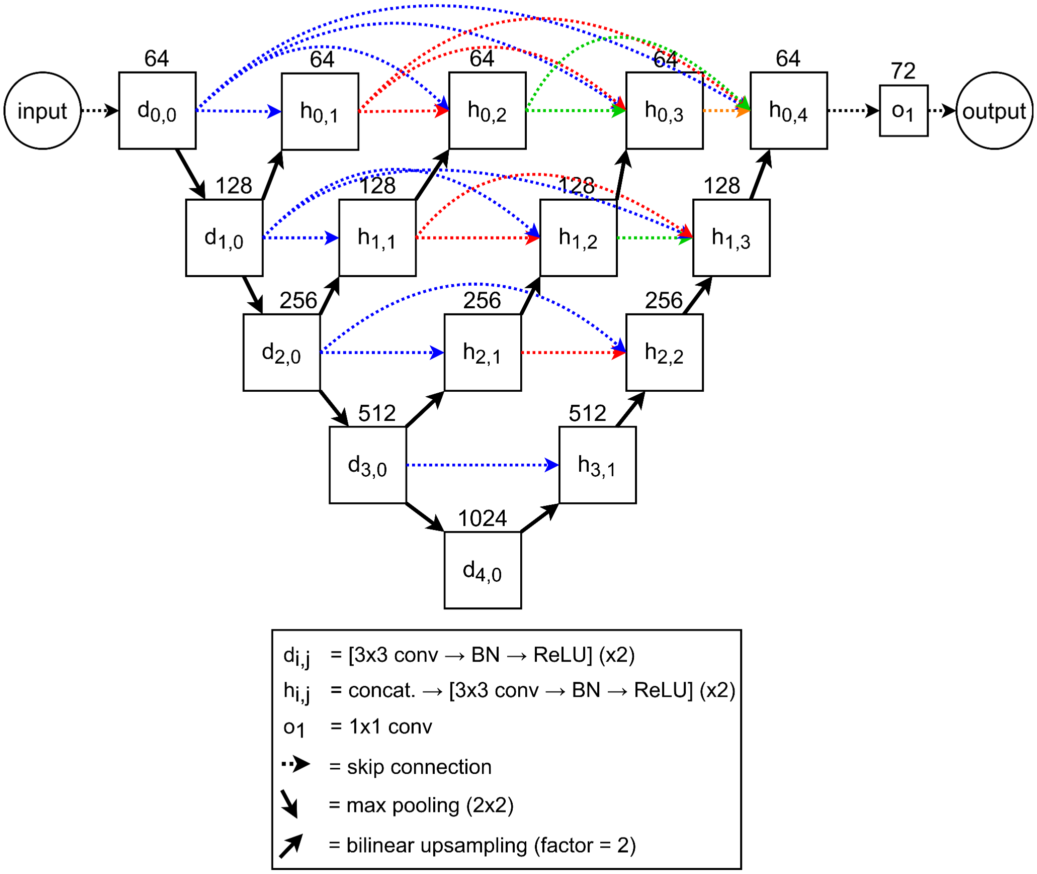

Supplementary Figure S3: UNet++ model architecture. All inputs to each $h_{i,j}$ are concatenated channel-wise into a single volume before the convolution operations are applied. Number above each node indicates the number of filters for all convolution operations within that node. All convolutions use 'same' padding. BN indicates batch normalisation, and ReLU indicates rectified linear unit activation.

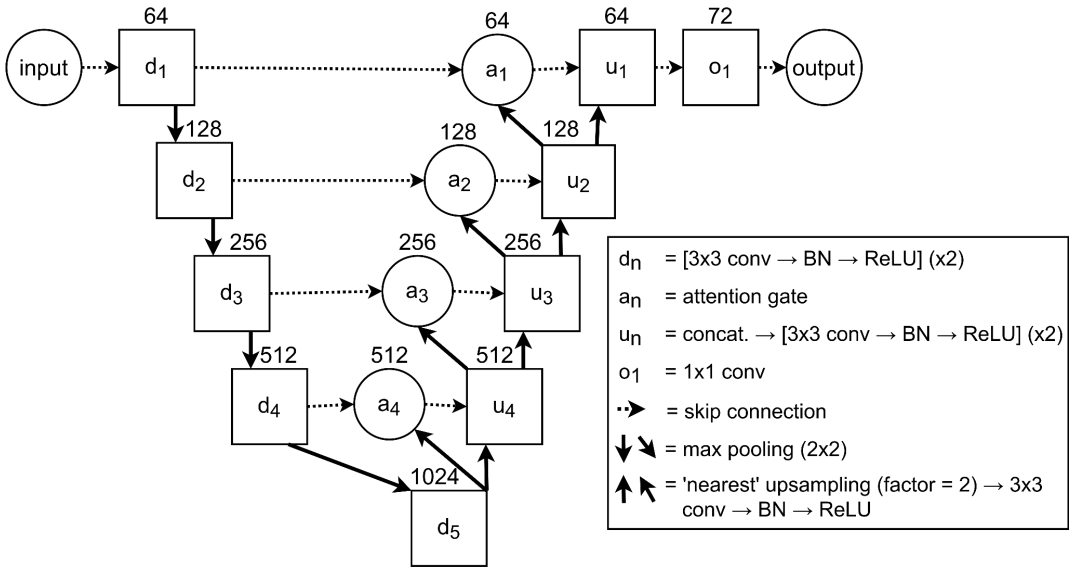

Supplementary Figure S4: Attention U-Net model architecture. Attention gate performs 1 × 1 convolution on each of its inputs, then sums the resulting volumes element-wise, followed by a ReLU, 1 × 1 convolution, batch normalisation, and application of a sigmoid function. All 3 × 3 convolutions use 'same' padding. Number above each node indicates the number of filters for all convolution operations within that node. BN indicates batch normalisation, and ReLU indicates rectified linear unit activation.

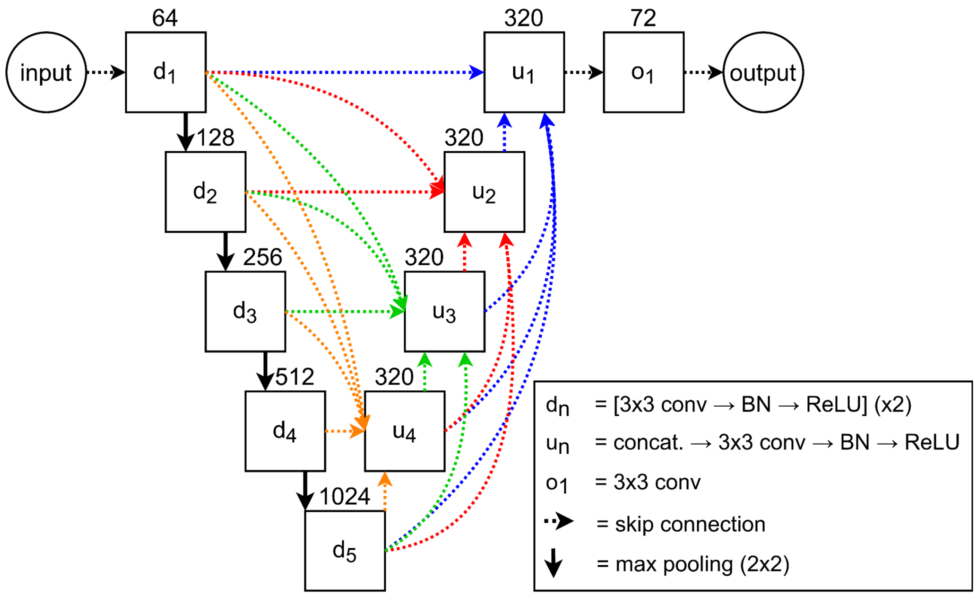

Supplementary Figure S5: UNet3+ architecture. All convolution operations are 3 × 3 with 'same' padding. Numbers above each node indicate the number of filters for all convolution operations within the node. All skip connections (except those between the input and d_1, u_1 and o_1, and o_1 and the output) scale their data via bilinear upsampling or max pooling to match the dimensions of the data at the destination node. This scaling is followed by a 3 × 3 convolution with 64 filters, which precedes the channel-wise concatenation in each u_n. BN indicates batch normalisation, and ReLU indicates rectified linear unit activation.

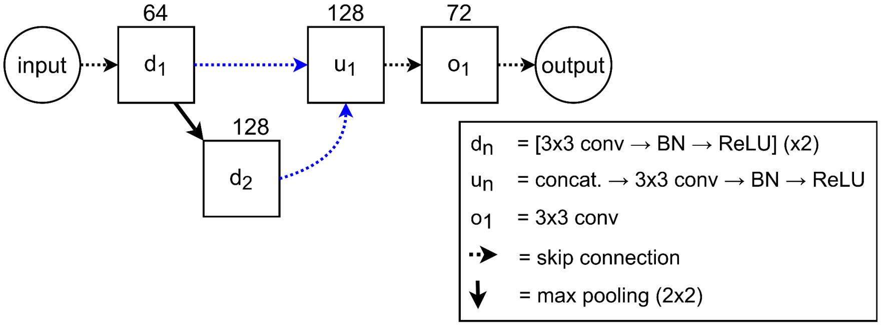

Supplementary Figure S6: UNet3+ architecture for model with depth 2. All convolution operations are 3 × 3 with 'same' padding. Numbers above each node indicate the number of filters for all convolution operations within the node. All skip connections (except those between the input and d_1, u_1 and o_1, and o_1 and the output) scale their data via bilinear upsampling or max pooling to match the dimensions of the data at the destination node. This scaling is followed by a 3 × 3 convolution with 64 filters, which precedes the channel-wise concatenation in each u_n. BN indicates batch normalisation, and ReLU indicates rectified linear unit activation.

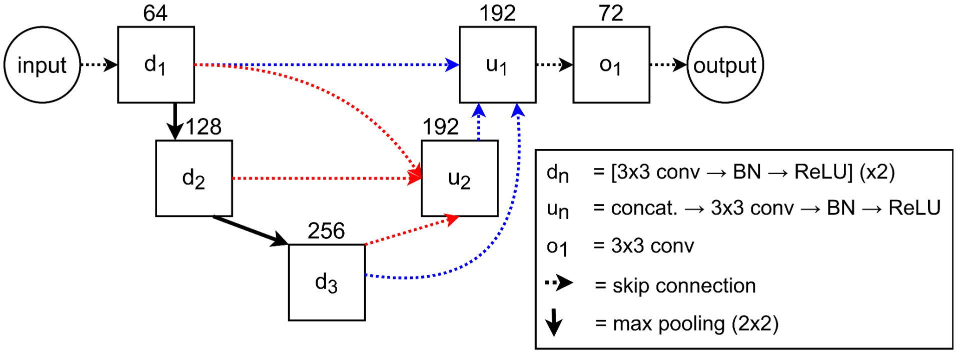

Supplementary Figure S7: UNet3+ architecture for model with depth 3. All convolution operations are 3 × 3 with 'same' padding. Numbers above each node indicate the number of filters for all convolution operations within the node. All skip connections (except those between the input and d_1, u_1 and o_1, and o_1 and the output) scale their data via bilinear upsampling or max pooling to match the dimensions of the data at the destination node. This scaling is followed by a 3 × 3 convolution with 64 filters, which precedes the channel-wise concatenation in each u_n. BN indicates batch normalisation, and ReLU indicates rectified linear unit activation.

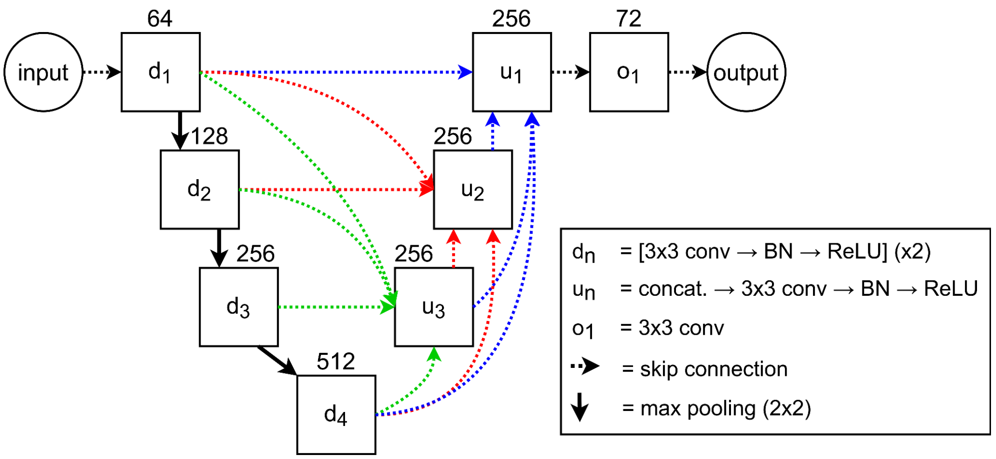

Supplementary Figure S8: UNet3+ architecture for model with depth 4. All convolution operations are 3 × 3 with ’same’ padding. Numbers above each node indicate the number of filters for all convolution operations within the node. All skip connections (except those between the input and d_1, u_1 and o_1, and o_1 and the output) scale their data via bilinear upsampling or max pooling to match the dimensions of the data at the destination node. This scaling is followed by a 3 × 3 convolution with 64 filters, which precedes the channel-wise concatenation in each u_n. BN indicates batch normalisation, and ReLU indicates rectified linear unit activation.

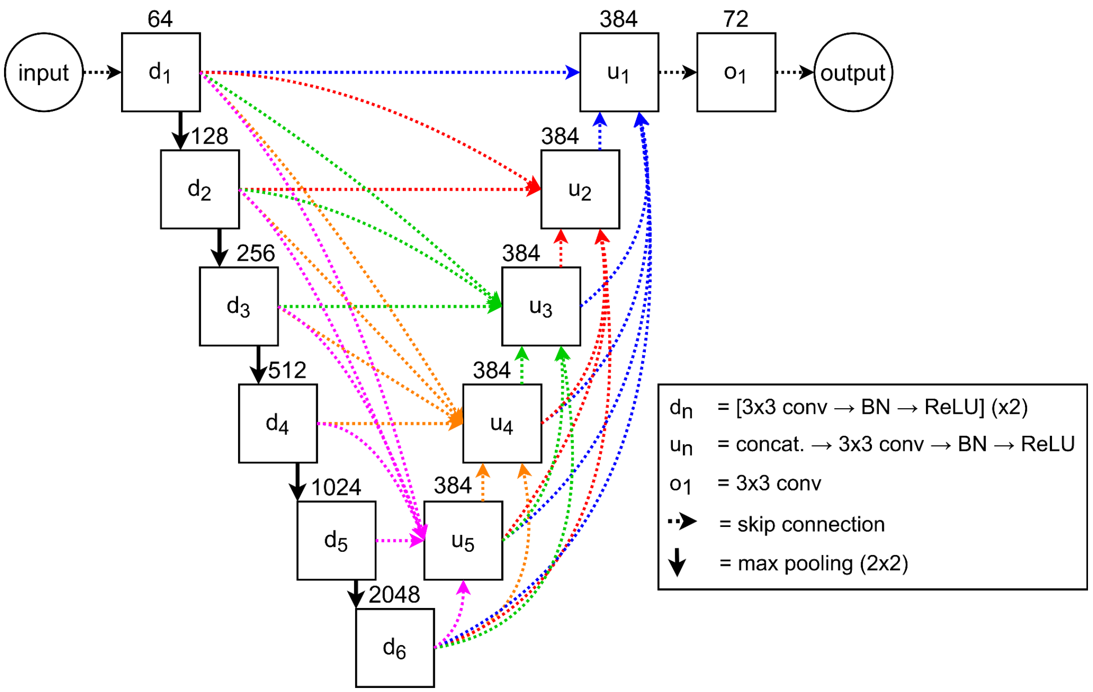

Supplementary Figure S9: UNet3+ architecture for model with depth 6. All convolution operations are 3 × 3 with ‘same’ padding. Numbers above each node indicate the number of filters for all convolution operations within the node. All skip connections (except those between the input and d_1, u_1 and o_1, and o_1 and the output) scale their data via bilinear upsampling or max pooling to match the dimensions of the data at the destination node. This scaling is followed by a 3 × 3 convolution with 64 filters, which precedes the channel-wise concatenation in each u_n. BN indicates batch normalisation, and ReLU indicates rectified linear unit activation.

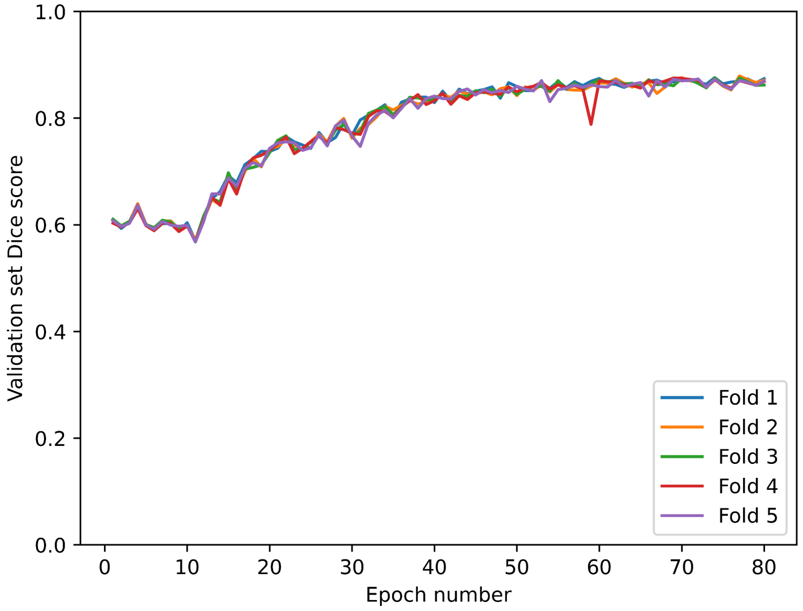

Supplementary Figure S10: Validation dice score for 80 epochs of NAS training.
